# “Almost-stable” matchings in the Hospitals / Residents problem with Couples

**DOI:** 10.1007/s10601-016-9249-7

**Published:** 2016-08-11

**Authors:** David F. Manlove, Iain McBride, James Trimble

**Affiliations:** School of Computing Science, University of Glasgow, Sir Alwyn Williams Building, Glasgow, G12 8QQ UK

**Keywords:** Most-stable matching, Blocking pair, Polynomial-time algorithm, NP-hardness, Integer programming model, Constraint programming model, Empirical evaluation

## Abstract

The Hospitals / Residents problem with Couples (hrc) models the allocation of intending junior doctors to hospitals where couples are allowed to submit joint preference lists over pairs of (typically geographically close) hospitals. It is known that a stable matching need not exist, so we consider min bp hrc, the problem of finding a matching that admits the minimum number of blocking pairs (i.e., is “as stable as possible”). We show that this problem is NP-hard and difficult to approximate even in the highly restricted case that each couple finds only one hospital pair acceptable. However if we further assume that the preference list of each single resident and hospital is of length at most 2, we give a polynomial-time algorithm for this case. We then present the first Integer Programming (IP) and Constraint Programming (CP) models for min bp hrc. Finally, we discuss an empirical evaluation of these models applied to randomly-generated instances of min bp hrc. We find that on average, the CP model is about 1.15 times faster than the IP model, and when presolving is applied to the CP model, it is on average 8.14 times faster. We further observe that the number of blocking pairs admitted by a solution is very small, i.e., usually at most 1, and never more than 2, for the (28,000) instances considered.

**Electronic supplementary material:**

The online version of this article (doi:10.1007/s10601-016-9249-7) contains supplementary material, which is available to authorized users.

## Introduction

### The Hospitals / Residents problem

The *Hospitals / Residents problem* (hr) [13] is a many-to-one allocation problem that models the assignment process involved in centralised matching schemes such as the National Resident Matching Program (NRMP) [42] which assigns graduating medical students to hospital posts in the USA. Analogous schemes exist in Canada [37] and Japan [39]. A similar process was used until recently to match medical graduates to Foundation Programme places in Scotland: the Scottish Foundation Allocation Scheme (SFAS) [19]. Moreover, similar matching schemes exist in the context of Higher Education admission in Hungary [4, 40], Spain [29], Turkey [3] and Ireland [38, 40]. The reader is referred to [40] for details of matching practices in a number of practical contexts throughout Europe.

An instance of hr consists of two sets of agents – a set $R= \{r_{1} ,{\ldots } r_{n_{1}}\}$ containing *residents* and a set $H= \{h_{1} ,{\ldots } h_{n_{2}}\}$ containing *hospitals*. Every resident expresses a linear preference over some subset of the hospitals, his *preference list*. The hospitals in a resident’s preference list are his *acceptable* partners; all other hospitals being *unacceptable*. Every hospital expresses a linear preference over those residents who find it acceptable. Further, each hospital *h*_*j*_∈*H* has a positive integral *capacity* *c*_*j*_, the maximum number of residents to which it may be assigned. A *matching* *M* is a set of acceptable resident-hospital pairs such that each resident appears in at most one pair and each hospital *h*_*j*_ belongs to at most *c*_*j*_ pairs. If (*r*_*i*_,*h*_*j*_)∈*M* then *r*_*i*_ is said to be *assigned* to *h*_*j*_, *M*(*r*_*i*_) denotes *h*_*j*_, and *r*_*i*_ is an *assignee* of *h*_*j*_. Given *r*_*i*_∈*R*, if *r*_*i*_ does not belong to any pair in *M* then *r*_*i*_ is said to be *unassigned*. Given *h*_*j*_∈*H*, we let *M*(*h*_*j*_) denote the set of assignees of *h*_*j*_ in *M*. Hospital *h*_*j*_ is *undersubscribed*, *full* or *oversubscribed* according as |*M*(*h*_*j*_)| is less than, equal to, or larger than *c*_*j*_, respectively.

Roth [31] argued that a key property to be satisfied by any matching *M* in an instance *I* of hr is *stability*, which ensures that *M* admits no *blocking pair* in *I*. Informally, such a pair comprises a resident *r*_*i*_ and a hospital *h*_*j*_, both of whom have an incentive to disregard their assignments (if any) and become matched to one another outside of *M*, undermining its integrity. A matching is *stable* if it admits no blocking pair. It is known that every instance of hr admits at least one stable matching, which can be found in time linear in the size of the instance [13].

### The Hospitals / Residents problem with Couples

The Hospitals / Residents problem with Couples (hrc) is a generalisation of hr that is important in practical applications because it models the case where some of the residents may apply jointly in couples, so that they may be matched to hospitals that are geographically close to one another. In order to ensure this, a couple submits a joint preference list over pairs of hospitals, rather than individual hospitals. Matching schemes for junior doctors such as the NRMP [42] allow couples to apply jointly, as do assignment processes in the US Navy [28, 34, 36] (for which hrc is an appropriate problem model), for example.

Formally, an instance *I* of hrc consists of a set $R=\{r_{1} ,{\ldots } r_{n_{1}}\}$ containing *residents* and a set $H=\{h_{1} ,{\ldots } h_{n_{2}}\}$ containing *hospitals*. The residents in *R* are partitioned into two sets, *S* and *S* ^′^. The set *S* consists of *single* residents and the set *S* ^′^ consists of those residents involved in *couples*. There is a set *C*={(*r*_*i*_,*r*_*j*_):*r*_*i*_,*r*_*j*_∈*S* ^′^} of *couples* such that each resident in *S* ^′^ belongs to exactly one pair in *C*.

Each single resident *r*_*i*_∈*S* expresses a linear preference order over some subset of the hospitals, his *acceptable* hospitals; all other hospitals being *unacceptable*. Each couple (*r*_*i*_,*r*_*j*_)∈*C* expresses a joint linear preference order over a subset *A* of *H*×*H* where (*h*_*p*_,*h*_*q*_)∈*A* represents the simultaneous assignment of *r*_*i*_ to *h*_*p*_ and *r*_*j*_ to *h*_*q*_. The hospital pairs in *A* represent those joint assignments that are *acceptable* to (*r*_*i*_,*r*_*j*_), all other joint assignments being *unacceptable*. Each hospital *h*_*j*_∈*H* expresses a linear preference order over those residents who find it acceptable, either as a single resident or as part of a couple, and as in the case of hr, each hospital *h*_*j*_∈*H* has a positive integral *capacity* *c*_*j*_.

A *matching* *M* in *I* is defined as in hr case, with the additional restriction that, for each couple (*r*_*i*_,*r*_*j*_)∈*C*, either both *r*_*i*_ and *r*_*j*_ appear in no pair of *M*, or else {(*r*_*i*_,*h*_*k*_),(*r*_*j*_,*h*_*l*_)}⊆*M* for some pair (*h*_*k*_,*h*_*l*_) that (*r*_*i*_,*r*_*j*_) find acceptable. In the former case, (*r*_*i*_,*r*_*j*_) are said to be *unassigned*, whilst in the latter case, (*r*_*i*_,*r*_*j*_) are said to be *jointly assigned* to (*h*_*k*_,*h*_*l*_). Given a resident *r*_*i*_∈*R*, the definitions of *M*(*r*_*i*_), *assigned* and *unassigned* are the same as for the hr case, whilst for a hospital *h*_*j*_∈*H*, the definitions of *assignees*, *M*(*h*_*j*_), *undersubscribed*, *full* and *oversubscribed* for hospitals are also the same as before.

We seek a *stable* matching, which guarantees that no resident and hospital, and no couple and pair of hospitals, have an incentive to deviate from their assignments and become assigned to each other outside of the matching. Roth [31] considered stability in the hrc context but did not define the concept explicitly. Whilst Gusfield and Irving [15] gave a formal definition of a blocking pair, it neglected to deal with the case that both members of a couple may wish to be assigned to the same hospital. A number of other stability definitions for hrc have since been given in the literature that address this issue (see [6] and [20, Section 5.3] for more details), including that of McDermid and Manlove [24], which we adopt in this paper. We repeat their definition again here for completeness.

### **Definition 1** (24)

Let *I* be an instance of hrc. A matching *M* is *stable* in *I* if none of the following holds:

1. There is a single resident *r*_*i*_ and a hospital *h*_*j*_, where *r*_*i*_ finds *h*_*j*_ acceptable, such that either *r*_*i*_ is unassigned in *M* or prefers *h*_*j*_ to *M*(*r*_*i*_), and either *h*_*j*_ is undersubscribed in *M* or prefers *r*_*i*_ to some member of *M*(*h*_*j*_).
2. There is couple (*r*_*i*_,*r*_*j*_) and a hospital *h*_*k*_ such that *either*
  - (*r*_*i*_,*r*_*j*_) prefers (*h*_*k*_,*M*(*r*_*j*_)) to (*M*(*r*_*i*_),*M*(*r*_*j*_)), and either *h*_*k*_ is undersubscribed in *M* or prefers *r*_*i*_ to some member of *M*(*h*_*k*_)∖{*r*_*j*_}*or*
  - (*r*_*i*_,*r*_*j*_) prefers (*M*(*r*_*i*_),*h*_*k*_) to (*M*(*r*_*i*_),*M*(*r*_*j*_)), and either *h*_*k*_ is undersubscribed in *M* or prefers *r*_*j*_ to some member of *M*(*h*_*k*_)∖{*r*_*i*_}.
3. There is a couple (*r*_*i*_,*r*_*j*_) and a pair of (not necessarily distinct) hospitals *h*_*k*_≠*M*(*r*_*i*_), *h*_*l*_≠*M*(*r*_*j*_) such that (*r*_*i*_,*r*_*j*_) finds (*h*_*k*_,*h*_*l*_) acceptable, and either (*r*_*i*_,*r*_*j*_) is unassigned or prefers the joint assignment (*h*_*k*_,*h*_*l*_) to (*M*(*r*_*i*_),*M*(*r*_*j*_)), and *either*
  - *h*_*k*_≠*h*_*l*_, and *h*_*k*_ (respectively *h*_*l*_) is either undersubscribed in *M* or prefers *r*_*i*_ (respectively *r*_*j*_) to at least one of its assignees in *M*; *or*
  - *h*_*k*_ = *h*_*l*_, and *h*_*k*_ has two free posts in *M*, i.e., *c*_*k*_−|*M*(*h*_*k*_)|≥2; *or*
  - *h*_*k*_ = *h*_*l*_, and *h*_*k*_ has one free post in *M*, i.e., *c*_*k*_−|*M*(*h*_*k*_)|=1, and *h*_*k*_ prefers at least one of *r*_*i*_,*r*_*j*_ to some member of *M*(*h*_*k*_); *or*
  - *h*_*k*_ = *h*_*l*_, *h*_*k*_ is full in *M*, *h*_*k*_ prefers *r*_*i*_ to some *r*_*s*_∈*M*(*h*_*k*_), and *h*_*k*_ prefers *r*_*j*_ to some *r*_*t*_∈*M*(*h*_*k*_)∖{*r*_*s*_}.
  A resident and hospital, or a couple and hospital pair, satisfying one of the above conditions, is called a *blocking pair* of *M* and is said to *block* *M*.

### Existing algorithmic results for hrc

An instance *I* of hrc need not admit a stable matching [31]. We call *I* *solvable* if it admits a stable matching, and *unsolvable* otherwise. Also an instance of hrc may admit stable matchings of differing sizes [2]. Further, the problem of deciding whether a stable matching exists in an instance of hrc is NP-complete, even in the restricted case where there are no single residents and each hospital has capacity 1 [25, 30]. The decision problem is also W[1]-hard [22] when parameterized by the number of couples.

In many practical applications of hrc the residents’ preference lists are short. Let (*α*,*β*,*γ*)-hrc denote the restriction of hrc in which each single resident’s preference list contains at most *α* hospitals, each couple’s preference list contains at most *β* pairs of hospitals and each hospital’s preference list contains at most *γ* residents. Biró et al. [7] showed that deciding whether an instance of (0,2,2)-hrc admits a stable matching is NP-complete.

Heuristics for hrc were described and compared experimentally by Biró et al. [5]. As far as exact algorithms are concerned, Biró et al. [7] gave an Integer Programming (IP) formulation for finding a maximum cardinality stable matching (or reporting that none exists) in an arbitrary instance of hrc and presented an empirical evaluation of an implementation of their model, showing that their formulation was capable of solving instances of the magnitude of those arising in the SFAS application. Further algorithmic results for hrc are given in [6, 20, 23].

### Most-stable matchings

Given that a stable matching need not exist in a given hrc instance *I*, a natural question to ask is whether there is some other matching that might be the best alternative amongst the matchings in *I*. Roth [32, 33] argued that instability in the outcome of an allocation process gives participants a greater incentive to circumvent formal procedures; it follows minimising the amount of instability might be a desirable objective. Eriksson and Häggström [11] suggested that the number of blocking pairs admitted by a matching is a meaningful way to measure its degree of instability.

Define *b* *p*(*M*) to be the set of blocking pairs relative to a matching *M* in *I*, and define a *most-stable matching* to be a matching *M* for which |*b* *p*(*M*)| is minimum, taken over all matchings in *I*. Clearly if *I* admits a stable matching *M*, then *M* is a most-stable matching in *I*. Let min bp hrc denote the problem of finding a most-stable matching, given an instance of hrc. Most-stable matchings have been studied from an algorithmic point of view in various matching problem contexts [1, 8, 9, 12, 16, 17] (see [20] for more details), including in humanitarian organisations [35]. Define (*α*,*β*,*γ*)-min bp hrc to be the restriction of min bp hrc to instances of (*α*,*β*,*γ*)-hrc.

### Contribution of this work

In Section 2 we show that (*∞*,1,*∞*)-min bp hrc is NP-hard and not approximable within $n_{1}^{1-\varepsilon }$, for any *ε*>0, unless P=NP (recall that *n*_1_ is the number of residents in a given instance). In this highly restricted case of min bp hrc, each couple finds only one hospital pair acceptable and each hospital has capacity 1 (*∞* refers to preference lists of unbounded length). We also show that (*∞*,*∞*,1)-min bp hrc and (2,1,2)-min bp hrc are solvable in polynomial time. These results help to narrow down the search for the boundary between polynomial-time solvable and NP-hard restrictions of min bp hrc (recall that (0,2,2)-min bp hrc is NP-hard [7]).

In Section 3 we present the first IP model for min bp hrc; indeed this model can be used to find a most-stable matching of maximum cardinality. This formulation extends our earlier IP model for hrc, presented in [7]. Then in Section 4 we present data from an empirical evaluation of an implementation of the IP model for min bp hrc applied to randomly-generated instances. We measure the mean solution time, mean size of a most-stable matching and mean number of blocking pairs admitted by a most-stable matching when varying (i) the number of residents, (ii) the number of couples, (iii) the number of hospitals and (iv) the lengths of the residents’ preference lists. Our main finding is that, over the 28,000 instances considered, the number of blocking pairs admitted by a most-stable matching is very small: it is usually at most 1, and never more than 2. This suggests that in a given hrc instance in practice, even if a stable matching does not exist, we may be able to find a matching with only a very small amount of instability.

Finally, in Section 5 we present the first Constraint Programming (CP) model for min bp hrc and evaluate its performance compared to the IP model over the instances used for the empirical analysis in Section 4. We observe that on average, the CP model is about 1.15 times faster than the IP model, and when presolving is applied to the CP model, it is on average 8.14 times faster.

### Related work

Drummond et al. [10] presented SAT and IP encodings of hrc and investigated empirically their performance, along with two earlier heuristics for hrc, on randomly-generated instances. Their main aim was to measure the time taken to find a stable matching or report that none exists, and the proportion of solvable instances. They found that the SAT encoding gave the fastest method and was generally able to resolve the solvability question for the highest proportion of instances. In another paper [27], the same authors conducted further empirical investigations on random instances using an extension of their SAT encoding to determine how many stable matchings were admitted, and whether a resident Pareto optimal stable matching existed. We remark that the results in [10, 27] are not directly comparable to ours, because the stability definition considered in those papers is slightly weaker than that given by Definition 1. See Section A of the online supplement for a discussion of this issue.

Hinder [18] presented an IP model for a general stable matching problem with contracts, which includes hrc as defined here, as a special case. He conducted an empircal study on randomly-generated instances, comparing the performance of the IP model, its LP relaxation and a previously-published heuristic. Hinder showed that the LP relaxation finds stable matchings (when they exist) with much higher probability than the heuristic, and with probability quite close to the true value given by the IP model. The IP model terminates surprisingly quickly when the number of residents belonging to a couple is 10 %, but it should be emphasised that in Hinder’s random instances, all hospitals have capacity 1. In such a case our IP/CP models would be much simpler and need not involve the constraints corresponding to stability criteria 3(b), 3(c) and 3(d) in Definition 1, thus our runtime results are not directly comparable to Hinder’s.

To the best of our knowledge there have been no previous CP models for hrc, though a CP model for hr was given in [21], extending an earlier CP model for the classical Stable Marriage problem, the 1-1 restriction of hr [14]. A detailed survey of CP models for stable matching problems is given in [20, Section 2.5].

Nguyen and Vohra [26] proved a remarkable result, namely that it is always possible to find a stable matching in an instance of hrc if the capacity of each hospital can be adjusted (up or down) by at most 4, with the total capacity of the hospitals increasing by at most 9.

## Complexity results for min bp hrc

In this section we present complexity and approximability results for min bp hrc in the case that preference lists of some or all of the agents are of bounded length. We begin with (*∞*,1,*∞*)-min bp hrc, the restriction in which each couple lists only one hospital pair on their preference list. Even in this highly restricted case, the problem of finding a most-stable matching is NP-hard and difficult to approximate. The proof of this result, given in Section B of the online supplement, begins by showing that, given an instance of (*∞*,1,*∞*)-hrc, the problem of deciding whether a stable matching exists is NP-complete. Then a gap-introducing reduction is given from this problem to (*∞*,1,*∞*)-min bp hrc.

### **Theorem 2**

(∞,1,∞)-min bp hrc *is NP-hard and not approximable within a factor of* $n_{1}^{1-\varepsilon }$ *, for any ε>0, unless P=NP, where n*_1_ *is the number of residents in a given instance. The result holds even if each hospital has capacity 1.*

We now turn to the case that hospitals’ lists are of bounded length. It will be helpful to introduce the notion of a *fixed assignment* in a given hrc instance *I*. This involves either (i) a resident-hospital pair (*r*_*i*_,*h*_*j*_) such that *h*_*j*_ is the first choice of *r*_*i*_, and *r*_*i*_ is among the first *c*_*j*_ choices of *h*_*j*_, or (ii) a pair comprising a couple (*r*_*i*_,*r*_*j*_) and a pair of hospitals (*h*_*p*_,*h*_*q*_) such that *h*_*p*_ (resp. *h*_*q*_) is the first choice of *r*_*i*_ (resp. *r*_*j*_), and *r*_*i*_ (resp. *r*_*j*_) is among the first *c*_*p*_ (resp. *c*_*q*_) choices of *h*_*p*_ (resp. *h*_*q*_). Clearly any stable matching must contain all the fixed assignments in *I*. By eliminating the fixed assignments iteratively, we arrive at the following straightforward result for (*∞*,*∞*,1)-hrc (the proofs of all the results stated in this section from this point onwards can be found in Section C of the online supplement).

### **Proposition 3**

*An instance I of* (∞,∞,1)-hrc *admits exactly one stable matching, which can be found in polynomial time.*

We now consider the (2,1,2)-hrc case. The process of *satisfying* a fixed assignment involves matching together the resident(s) and hospital(s) involved, deleting the agents themselves (and removing them from the remaining preference lists). This may uncover further fixed assignments, which themselves can be satisfied. Once this process terminates, we say that all fixed assignments have been *iteratively satisfied*. Let *I* be the (2,1,2)-hrc instance that remains. It turns out that *I* has a special structure, as the following result indicates.

### **Lemma 4**

**Fig. 1:**
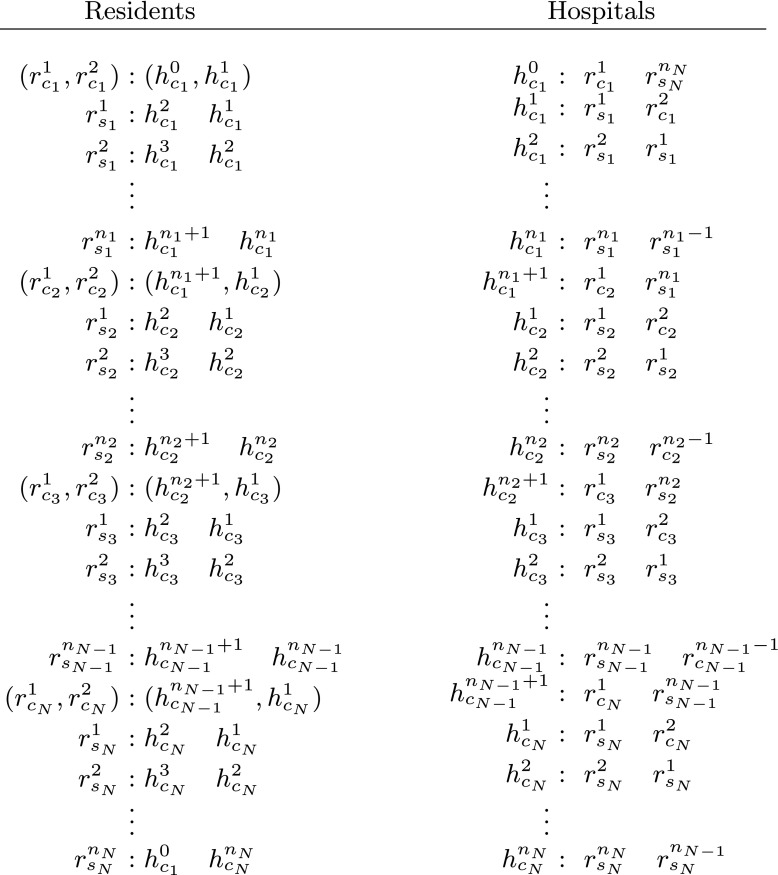
An instance of (2,1,2)-hrc containing an arbitrary number of couples and an arbitrary number of residents that has no unsatisfied fixed assignments. Here residents with a subscript *s* are single residents, whilst those with a subscript *c* belong to couples. The structure of this instance is described in more detail in Section C of the online supplement

*An arbitrary instance of (2,1,2)* -hrc *involving at least one couple and in which all fixed assignments have been iteratively satisfied must be constructed from sub-instances of the form shown in* Fig. 1 *, in which all of the hospitals have capacity 1.*

It is then straightforward to find a most-stable matching in each such sub-instance.

### **Lemma 5**

*Let I* ^′^ *be an instance of (2,1,2)* -hrc *of the form shown in Fig.* 1 *. If I* ^′^ *has an even number of couples then I* ^′^ *admits a stable matching M. Otherwise I* ^′^ *admits a matching M such that |bp(M)|=1 in I* ^′^.

Using Lemmas 4 and 5, it follows that we can find a most-stable matching in an instance *I* of (2,1,2)-hrc as follows. Assume that *M*_0_ is the matching in *I* in which all fixed assignments have been iteratively satisfied, and assume that the corresponding deletions have been made from the preference lists in *I*, yielding instance *I* ^′^. Lemma 4 shows that *I* ^′^ is a union of disjoint sub-instances *I*_1_,*I*_2_,…,*I*_*t*_, where each *I*_*j*_ is of the form shown in shown in Fig. 1 (1≤*j*≤*t*). Let *j* (1≤*j*≤*t*) be given and let *N*_*j*_ be the number of couples in *I*_*j*_. Lemma 5 implies that, if *N*_*j*_ is even, we may find a stable matching *M*_*j*_ in *I*_*j*_, otherwise we may find a matching *M*_*j*_ in *I*_*j*_ such that |*b* *p*(*M*_*j*_)|=1 in *I*_*j*_. It follows that $M=\cup _{j=0}^{t} M_{j}$ is a most-stable matching in *I*. This leads to the following result.

### **Theorem 6**

*(2,1,2)* -min bp hrc *is solvable in polynomial time.*

It remains open to resolve the complexity of (*p*,1,*q*)-hrc for constant values of *p* and *q* where max{*p*,*q*}≥3.

## An integer programming formulation for min bp hrc

In this section we describe our IP model for min bp hrc, which extends the earlier IP model for hrc presented in [7] (we discuss relationships between the two models at the end of this section). Let *I* be an instance of hrc where $R=\{r_{1},r_{2},\dots ,r_{n_{1}}\}$ is the set of residents and $H=\{h_{1},h_{2},\dots ,h_{n_{2}}\}$ is the set of hospitals; we will denote by *J* the IP model corresponding to *I*. To streamline the exposition we will only present some of the constraints in *J*; the full description of *J* is contained in Section D of the online supplement.

The IP model *J* is based on modelling the various types of blocking pairs that might arise according to Definition 1, and allowing them to be counted by imposing a series of linear inequalities. The variables are defined for each resident, whether single or a member of a couple, and for each element on his preference list (with the possibility of being unassigned). A further consistency constraint ensures that each member of a couple obtains hospitals from the same pair in their list, if assigned. A suitable objective function then enables the number of blocking pairs to be minimised. Subject to this, we may also maximise the size of the constructed matching.

### Notation

We first define some required notation in *I*. Without loss of generality, suppose residents *r*_1_,*r*_2_…*r*_2*c*_ are in couples. Thus $r_{2c+1}, r_{2c+2}{\ldots } r_{n_{1}}$ comprise the single residents. Again, without loss of generality, suppose that the couples are (*r*_2*i*−1_,*r*_2*i*_)(1≤*i*≤*c*). A crucial component of the IP model is a mapping between the joint preference list of a couple $\mathcal C_{i} = (r_{2i-1}, r_{2i})$ and individual preference lists for *r*_2*i*−1_ and *r*_2*i*_. Suppose that the joint preference list of $\mathcal C_{i}$ is

$$\mathcal C_{i} ~ : (h_{\alpha_{1}}, h_{\beta_{1}}),(h_{\alpha_{2}}, h_{\beta_{2}}){\ldots} (h_{\alpha_{l}}, h_{\beta_{l}}).$$

From this list we say that $h_{\alpha _{1}}, h_{\alpha _{2}}{\ldots } h_{\alpha _{l}}$ and $h_{\beta _{1}}, h_{\beta _{2}}{\ldots } h_{\beta _{l}}$ are the *individual* preference lists for *r*_2*i*−1_ and *r*_2*i*_ respectively. Note that a given hospital *h*_*j*_ may appear more than once in the individual preference list of a resident belonging to a couple.

For a resident *r*_*i*_∈*R* (whether single or a member of a couple), let *l*(*r*_*i*_) denote the length of a resident *r*_*i*_’s individual preference list. Moreover let *pref* (*r*_*i*_,*p*) denote the hospital at position *p* of *r*_*i*_’s individual preference list.

For a hospital *h*_*j*_∈*H*, let *l*(*h*_*j*_) denote the length of *h*_*j*_’s preference list over individual residents. For an acceptable resident-hospital pair (*r*_*i*_,*h*_*j*_), let *rank* (*h*_*j*_,*r*_*i*_) = *q* denote the rank that hospital *h*_*j*_ assigns resident *r*_*i*_, where 1≤*q*≤*l*(*h*_*j*_). Thus, *rank* (*h*_*j*_,*r*_*i*_) is equal to the number of residents that *h*_*j*_ prefers to *r*_*i*_ plus 1. Further, for each *j*(1≤*j*≤*n*_2_) and *q*(1≤*q*≤*l*(*h*_*j*_)), let the set *R*(*h*_*j*_,*q*) contain resident-position pairs (*r*_*i*_,*p*) such that *r*_*i*_∈*R* is assigned a rank of *q* by *h*_*j*_ and *h*_*j*_ is in position *p*(1≤*p*≤*l*(*r*_*i*_)) on *r*_*i*_’s individual list. Hence

$$R(h_{j}, q) = \{(r_{i}, p)\in R \times \mathbb{Z} : \textit{rank}(h_{j}, r_{i}) = q \wedge 1\leq p\leq l(r_{i})\wedge \textit{pref}(r_{i}, p) = h_{j}\}.$$

### Variables in the IP model

For each *i*(1≤*i*≤*n*_1_) and *p*(1≤*p*≤*l*(*r*_*i*_)), *J* has a variable *x*_*i*,*p*_∈{0,1} such that *x*_*i*,*p*_=1 if and only if *r*_*i*_ is assigned to his *p* ^*th*^-choice hospital. Also, for each *i* (1≤*i*≤*n*_1_) and *p* = *l*(*r*_*i*_)+1, *J* has a variable *x*_*i*,*p*_∈{0,1} such that *x*_*i*,*p*_=1 if and only if *r*_*i*_ is unassigned. Let *X*={*x*_*i*,*p*_:1≤*i*≤*n*_1_∧1≤*p*≤*l*(*r*_*i*_)+1}.

*J* also contains variables *𝜃*_*i*,*p*_∈{0,1} for each *i*(1≤*i*≤*n*_1_) and *p*(1≤*p*≤*l*(*r*_*i*_)). The intuitive meaning of a variable *𝜃*_*i*,*p*_ is that *𝜃*_*i*,*p*_=1 if and only if resident *r*_*i*_ is involved in a blocking pair with the hospital at position *p* on his individual preference list, either as a single resident or as part of a couple.

### Constraints in the IP model

We firstly add constraints to *J* which force every variable to be binary valued. Next we ensure that matching constraints are satisfied, as follows. As each resident *r*_*i*_∈*R* is assigned to exactly one hospital or is unassigned (but not both), ${\sum }_{p=1}^{l(r_{i})+1} x_{i,p}=1$ must hold for all *i*(1≤*i*≤*n*_1_). Similarly, since a hospital *h*_*j*_ may be assigned at most *c*_*j*_ residents, *x*_*i*,*p*_=1 where *pref* (*r*_*i*_,*p*) = *h*_*j*_ for at most *c*_*j*_ residents, and hence for all *j*(1≤*j*≤*n*_2_), ${\sum }_{i=1}^{n_{1}}{\sum }_{p=1}^{l(r_{i})} \{x_{i,p} \in X :\,\, $ *pref* (*r*_*i*_,*p*) = *h*_*j*_}≤*c*_*j*_ must hold.

For each couple (*r*_2*i*−1_,*r*_2*i*_), *r*_2*i*−1_ is unassigned if and only if *r*_2*i*_ is unassigned, and *r*_2*i*−1_ is assigned to the hospital in position *p* in their individual list if and only if *r*_2*i*_ is assigned to the hospital in position *p* in their individual list. Thus for all *i*(1≤*i*≤*c*) and *p*(1≤*p*≤*l*(*r*_2*i*−1_)+1), *x*_2*i*−1,*p*_ = *x*_2*i*,*p*_ must hold,

The remaining constraints in *J* allow the number of blocking pairs of a given matching to be counted. Each such constraint deals with a specific type of blocking pair that satisfies a given part of Definition 1. It allows a blocking pair to exist involving either (i) a single resident *r*_*i*_ with the hospital at some position *p* on his list, or (ii) a couple (*r*_2*i*−1_,*r*_2*i*_) with the hospital pair at some position *p* on their joint list, if and only if *𝜃*_*i*,*p*_=1. We illustrate the construction of *J* by giving the constraint corresponding to so-called “Type 1” blocking pairs, involving involve single residents, where Condition 1 of Definition 1 is satisfied. The other constraints may be dealt with in a similar fashion – see Section D of the online supplement for further details.

### Type 1 blocking pairs

In a matching *M* in *I*, if a single resident *r*_*i*_∈*R* is unassigned or has a worse partner than some hospital *h*_*j*_∈*H* where *pref* (*r*_*i*_,*p*) = *h*_*j*_ and *rank* (*h*_*j*_,*r*_*i*_) = *q* then *h*_*j*_ must be fully subscribed with better partners than *r*_*i*_, for otherwise (*r*_*i*_,*h*_*j*_) blocks *M*. Hence if *r*_*i*_ is unassigned or has worse partner than *h*_*j*_, i.e., $\sum \limits _{p^{\prime }=p+1}^{l(r_{i})+1} x_{i,p^{\prime }}=1$, and *h*_*j*_ is not fully subscribed with better partners than *r*_*i*_, i.e., $\sum \limits _{q^{\prime }=1}^{q-1} \{x_{i^{\prime },p^{\prime \prime }} \in X : (r_{i^{\prime }}, p^{\prime \prime }) \in R(h_{j}, q^{\prime })\} < c_{j}$, then we require *𝜃*_*i*,*p*_=1 to count this blocking pair. Thus, for each *i*(2*c*+1≤*i*≤*n*_1_) and *p*(1≤*p*≤*l*(*r*_*i*_)) we obtain the following constraint where *pref* (*r*_*i*_,*p*) = *h*_*j*_ and *rank* (*h*_*j*_,*r*_*i*_) = *q*:

$$c_{j} \left( \left( \sum\limits_{p^{\prime}=p+1}^{l(r_{i})+1} x_{i,p^{\prime}}\right) - \theta_{i,p}\right) \leq \sum\limits_{q^{\prime}=1}^{q-1} \{x_{i^{\prime },p^{\prime \prime}} \in X : (r_{i^{\prime }}, p^{\prime \prime}) \in R(h_{j}, q^{\prime })\}.$$

### Objective functions in the IP model

A maximum cardinality most-stable matching *M* is a matching of maximum cardinality, taken over all most-stable matchings in *I*. To compute a maximum most-stable matching in *J*, we apply two objective functions in sequence.

First we find an optimal solution in *J* that minimises the number of blocking pairs. To this end we apply the objective function $\min \sum \limits _{i=1}^{n_{1}} \sum \limits _{p=1}^{l(r_{i})} \theta _{i,p}$.

The matching *M* corresponding to an optimal solution in *J* will be a most-stable matching in *I*. Let *k*=|*b* *p*(*M*)|. Now we seek a maximum cardinality matching in *I* with at most *k* blocking pairs. Thus we add the following constraint to *J*, which ensures that, when maximising on cardinality, any solution also has at most *k* blocking pairs: $\sum \limits _{i=1}^{n_{1}} \sum \limits _{p=1}^{l(r_{i})} \theta _{i,p} \leq k$.

The final step is to maximise the size of the matching, subject to the matching being most-stable. This involves optimising for a second time, this time using the following objective function: $\max \sum \limits _{i=1}^{n_{1}} \sum \limits _{p=1}^{l(r_{i})} x_{i,p}$.

The following result, which establishes the correctness of the IP formulation, is proved in Section D of the online supplement.

### **Theorem 7**

*Given an instance I of* min bp hrc, *let J be the corresponding IP model as defined above. A maximum cardinality most-stable matching in I is exactly equivalent to an optimal solution to J.*

We remark that the IP model presented in this section develops the earlier model for hrc [7] with the addition of the *𝜃*_*i*,*p*_ variables. There are similarities between the constraints (with these variables omitted) when comparing the two models. However in the hrc model [7] essentially all stability constraints had to be satisfied, whereas in the min bp hrc model a blocking pair is allowed at the expense of a *𝜃*_*i*,*p*_ variable having value 1, which allows the number of blocking pairs to be counted. Suitable placement of the *𝜃*_*i*,*p*_ variables within the constraints from the hrc model allows this condition on the *𝜃*_*i*,*p*_ variables to be enforced.

## Empirical results from the IP model for min bp hrc

In this section we present data from an empirical evaluation of an implementation of the IP model for finding a maximum cardinality most-stable matching in an instance of min bp hrc. We considered the following properties for randomly-generated hrc instances: the time taken to find a maximum cardinality most-stable matching, the size of a maximum cardinality most-stable matching and the number of blocking pairs admitted by a most-stable matching. We show how these properties varied as we modified the number of residents, the percentage of residents involved in couples, the number of hospitals and the lengths of residents’ preference lists in the constructed instances.

### Methodology

We ran all the experiments on an implementation of the IP model using the CPLEX 12.4 Java Concert API applied to randomly-generated instances of hrc.^1^ In these instances, the preference lists of residents and hospitals were constructed to take into account of the fact that, in reality, some hospitals and residents are more popular than others, respectively. Typically, the most popular hospital in the SFAS context had 5-6 times as many applicants as the least popular, and the numbers of applicants to the other hospitals were fairly uniformly distributed between the two extremes. Our constructed instances reflected this real-world behaviour. For more details about the construction of the instances and the correctness testing methodology, the reader is referred to [23, Chapters 6,7].

All experiments were carried out on a desktop PC with an Intel i5-2400 3.1Ghz processor with 8Gb of memory running Windows 7. To find a most-stable matching in an instance *I* of hrc we applied the following procedure. We first used the hrc IP implementation presented in [7] to find a maximum cardinality stable matching *M* in *I* if one exists. Clearly, if *I* is solvable then *M* is a maximum cardinality most-stable matching. However, if *I* was found to be unsolvable, we applied the min bp hrc IP model to *I*. In this case we applied a lower bound of 1 to the number of blocking pairs in a most-stable matching in *I* since we knew that no stable matching existed. All instances were allowed to run to completion. We remark that the min bp hrc model appears to be much more difficult to solve than the hrc model presented in [7], and thus the largest instances sizes considered here are smaller than the largest ones generated in the experimental evaluation in [7].

### Experiment 1

**Fig. 2:**
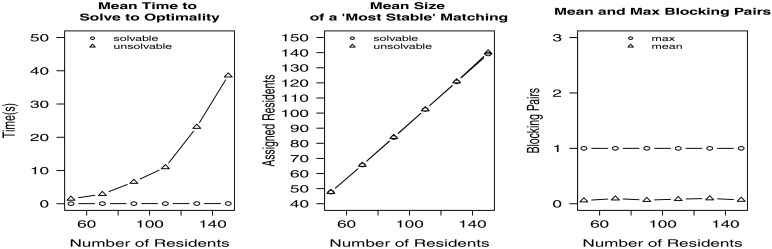
Empirical results for Experiment 1

In the first experiment we increased the number of residents while maintaining a constant ratio of couples, hospitals and posts to residents. For various values of *x*(50≤*x*≤150) in increments of 20, 1000 randomly generated instances were created containing *x* residents, 0.1*x* couples (and hence 0.8*x* single residents) and 0.1*x* hospitals with *x* available posts that were randomly distributed amongst the hospitals. Each resident’s preference list contained a minimum of 3 and a maximum of 5 hospitals. Figure 2 (and indeed all the figures in this section) shows the mean time taken to find a maximum cardinality most-stable matching, the mean size of a maximum cardinality most-stable solution (in each case over both solvable and unsolvable instances), and the mean and maximum number of blocking pairs admitted by most-stable matchings.

The results show that the time taken to find an optimal solution increases with *x*, with the min bp hrc formulation being more difficult to solve in general than the hrc formulation. The mean size of an optimal solution increases with *x* for both solvable and unsolvable instances (it is around 95 % of *x* for *x*=50, decreasing to around 93 % of *x* for *x*=150, with the optimal matching size for unsolvable instances being very slightly larger than that for solvable instances). Perhaps most interestingly, the maximum number of blocking pairs was 1, with the mean at most 0.1, and the mean number of unsolvable instances being 77.

### Experiment 2

**Fig. 3:**
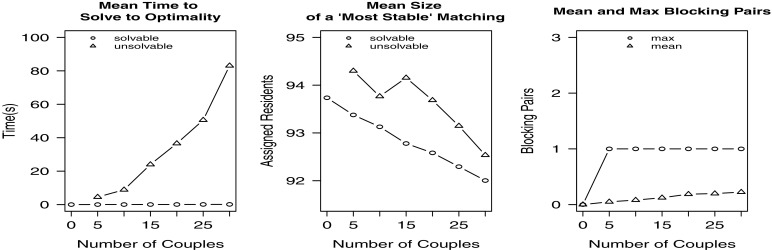
Empirical results for Experiment 2

In our second experiment we increased the percentage of residents involved in couples while maintaining the same numbers of residents, hospitals and posts. For various values of *x*(0≤*x*≤30) in increments of 5, 1000 randomly generated instances were created containing 100 residents, *x* couples (and hence 100−2*x* single residents) and 10 hospitals with 100 available posts that were unevenly distributed amongst the hospitals. Each resident’s preference list contained a minimum of 3 and a maximum of 5 hospitals. The results for all values of *x* are displayed in Fig. 3.

The results show that the time taken to find an optimal solution increases with *x*; again the min bp hrc formulation is more difficult to solve in general than the hrc formulation. The mean size of an optimal solution decreases with *x* for both solvable and unsolvable instances; again the optimal matching size for unsolvable instances is slightly larger than that for solvable instances. As for Experiment 1, the maximum number of blocking pairs was 1, with the number of unsolvable instances increasing from 50 for *x*=5 to 224 for *x*=30.

### Experiment 3

**Fig. 4:**
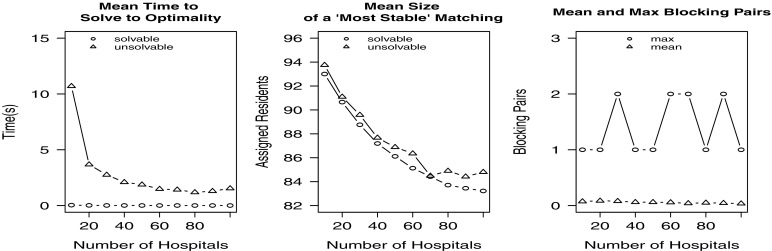
Empirical results for Experiment 3

In our third experiment we increased the number of hospitals in the instance while maintaining the same numbers of residents, couples and posts. For various values of *x*(10≤*x*≤100) in increments of 10, 1000 randomly generated instances were created containing 100 residents, 10 couples (and hence 80 single residents) and *x* hospitals with 100 available posts that were unevenly distributed amongst the hospitals. Each resident’s preference list contained a minimum of 3 and a maximum of 5 hospitals. The results for all values of *x* are displayed in Fig. 4.

The results show that the time taken to find an optimal solution decreases with *x*; again the min bp hrc model solution time is slower than that for the hrc model. Clearly the problem is becoming less constrained as the number of hospitals increases. Also the mean size of an optimal solution decreases with *x* for both solvable and unsolvable instances; again the optimal matching size for unsolvable instances is slightly larger than that for solvable instances. This time the maximum number of blocking pairs was 2, with the mean number of blocking pairs decreasing from 0.08 for *x*=20 to 0.04 for *x*=100.

### Experiment 4

**Fig. 5:**
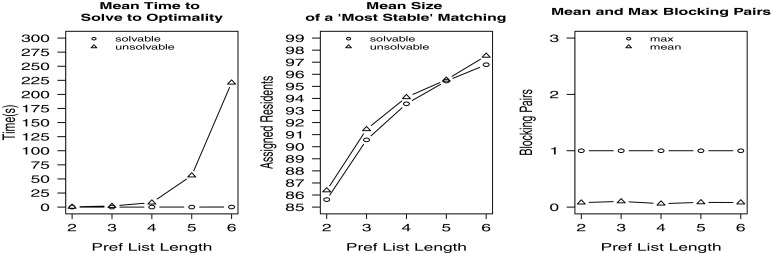
Empirical results for Experiment 4

In our last experiment, we increased the length of the individual preference lists for the residents in the instance while maintaining the same numbers of residents, couples, hospitals and posts. For various values of *x*(2≤*x*≤6), 1000 randomly generated instances were created containing 100 residents, 10 couples (and hence 80 single residents) and 10 hospitals with 100 available posts that were unevenly distributed amongst the hospitals. Each resident’s preference list contained exactly *x* hospitals. The results for all values of *x* are displayed in Fig. 5.

The results show that increasing the preference list length makes the problem harder to solve; again the min bp hrc model is slower to solve than the hrc model. Also the mean size of an optimal solution increases with *x* for both solvable and unsolvable instances as more options become available in the preference lists (from 86.4 for *x*=2 to 97.5 for *x*=6 in the case of unsolvable instances); again the optimal matching size for unsolvable instances is slightly larger than that for solvable instances. The maximum number of blocking pairs was 1, with the mean at most 0.1, and the mean number of unsolvable instances being 81.

### Discussion

The results presented in this section suggest that, even as we increase the number of residents or hospitals, the percentage of residents involved in a couple or the length of the residents’ preference lists, the number of blocking pairs admitted by a most-stable matching is very low. For most of the 28,000 instances generated in our experimental evaluation, the most-stable matchings found admitted at most 1 blocking pair, and the maximum number of blocking pairs admitted by any most-stable matching was never more than 2. These findings are essentially consistent with the results of Nguyen and Vohra [26], who showed that an unsolvable hrc instance only requires a small amount of perturbation in order to become solvable. Further empirical investigation is required to determine whether this behaviour is replicated for larger hrc instance sizes.

## A constraint programming model for min bp hrc

In addition to the IP model, we designed a Constraint Programming model for min bp hrc and implemented this using the MiniZinc constraint modelling language.

We assume that residents’ preference lists are given by integer variables *rpref* [*i*][*j*], which play a similar role to *pref* (*r*_*i*_,*j*) in the IP model, and that hospitals’ ranking arrays are given by integer variables *hrank* [*h*,*i*], which are analogous to *rank* (*h*_*j*_,*r*_*i*_) in the IP model. The lengths of the preference lists of a resident *r*_*i*_ and a hospital *h*_*j*_ are given by *rpref*_*len* [*i*] and *hpref*_*len* [*j*] respectively. The capacity of a hospital *h*_*j*_ is given by *h* *o* *s* *p* _*c* *a* *p*[*j*].

For each single resident *r*_*i*_, the model includes an integer variable *single*_*pos* [*i*] with domain (1,…,*l*(*r*_*i*_)+1), where *l*(*r*_*i*_) is the value of *rpref*_*len* [*i*], which takes the value *j* if *r*_*i*_ is assigned her *j*th-choice hospital, or *l*(*r*_*i*_)+1 if *r*_*i*_ is unassigned. For each couple *i*, we include an integer variable *c* *o* *u* *p* _*p* *o* *s*[*i*] with a similar interpretation.

Each single resident’s *single*_*pos* [*i*] variable is channelled to an array of *l*(*r*_*i*_) boolean variables *single*_*assigned* [*i*], such that *single*_*assigned* [*i*][*j*]= true if and only if *single*_*pos* [*i*] = *j*, and a variable *single*_*unassigned* [*i*], such that *single*_*unassigned* [*i*]= true if and only if *single*_*pos* [*i*] = *l*(*r*_*i*_)+1. Similarly, we have boolean *coup*_*assigned* and *coup*_*unassigned* variables for each couple.

For each hospital *i*, and each position *j* on hospital *i*’s preference list, we have a boolean variable *hosp*_*assigned* [*i*][*j*] which is true if and only if hospital *i* is assigned its *j*th-choice resident. We include a constraint to ensure that *hosp*_*assigned* [*i*][*j*]= true if and only if a corresponding *single*_*assigned* or *coup*_*assigned* variable is also true. Furthermore, each hospital has a linear inequality constraint to ensure that its capacity is not exceeded.

For each position on the preference list of a single resident or couple, we create a boolean variable *single*_*bp* [*i*][*j*] or *coup*_*bp* [*i*][*j*] indicating whether the resident or couple, along with their *j*th-choice hospital, constitutes a blocking pair. For each type of blocking pair, we define a set of constraints and then give some brief intuition.

### **Type 1 blocking pairs**


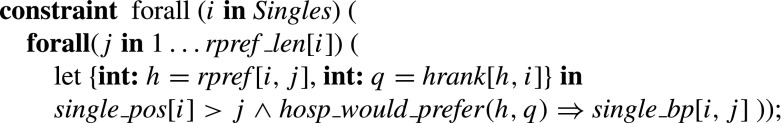


The *hosp*_*would*_*prefer* predicate for a hospital *h* and a position *q* on the preference list of *h* takes the value true if and only if *h* has fewer than *hosp*_*cap* [*h*] assigned residents in positions strictly preferable to position *q* on its preference list. (Note the redundancy in this predicate: all we actually need is the first *s* *u* *m*(… )(… )<*hosp*_*cap* [*h*] constraint; the *s* *u* *m*(… )(… )>0 constraint improves propagation.) 
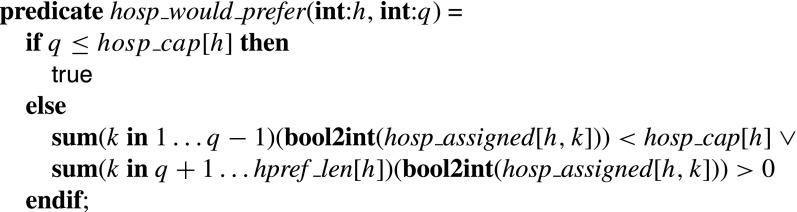


The constraint for Type 1 blocking pairs thus sets *single*_*bp* [*i*,*j*] to true if and only if *r*_*i*_ is unassigned or prefers *h* to his partner, and *h* is undersubscribed or prefers *r*_*i*_ at least one of its assignees, where *h* = *rpref* [*i*,*j*].

### **Type 2a/b blocking pairs**


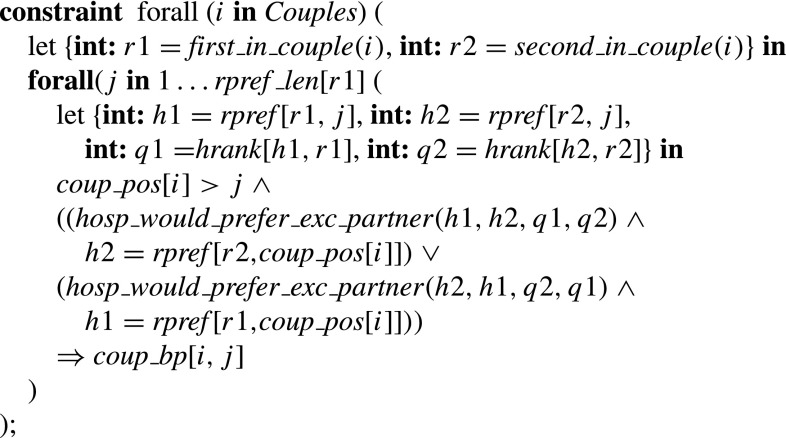


The *hosp*_*would*_*prefer*_*exc*_*partner* predicate on inputs *h*1, *h*2, *q*1, *q*2 (where *h*1, *h*2 are hospitals and *q*1, *q*2 are positions on their preference lists respectively) takes the value true if and only if (a) *h*1 = *h*2, *q*1<*q*2 and the number of *h*1’s assignees that it prefers to its *q*1th choice is less than *h* *o* *s* *p* _*c* *a* *p*[*h*1]−1, or (b) *h*1≠*h*2 or *q*1>*q*2 and the number of *h*1’s assignees that it prefers to its *q*1th choice is less than *h* *o* *s* *p* _*c* *a* *p*[*h*1]. 
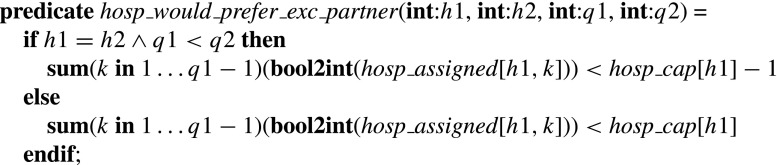


The constraint for Type 2a/b blocking pairs thus sets *coup*_*bp* [*i*,*j*] to true if and only if couple (*r*1,*r*2) prefer hospital pair (*h*1,*h*2) to their joint assignment (*h*3,*h*4), where *either*

- *h*2 = *h*4 and either *h*1 is undersubscribed or prefers *r*1 to at least one assignee that is not *r*2 (if *r*2 is assigned to *h*1) *or*
- *h*1 = *h*3 and either *h*2 is undersubscribed or prefers *r*2 to at least one assignee that is not *r*1 (if *r*1 is assigned to *h*2).

### **Type 3a blocking pairs**


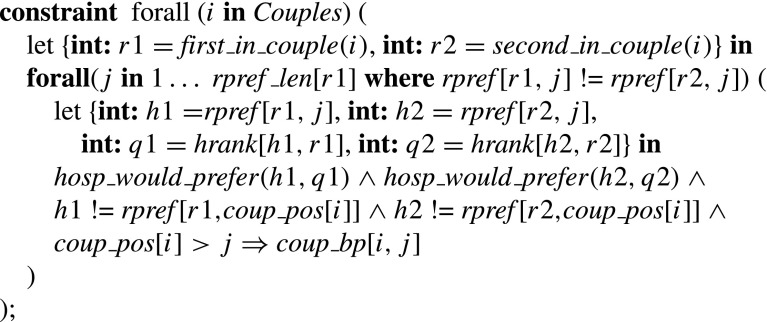


The constraint for Type 3a blocking pairs thus sets *coup*_*bp* [*i*,*j*] to true if and only if couple (*r*1,*r*2) are unassigned or prefer (*h*1,*h*2) to their joint assignment, whilst for each *k*∈{1,2}, *hk* is undersubscribed or prefers *rk* to at least one of its assignees, where (*r*1,*r*2) is the *i*th couple and (*h*1,*h*2) is the hospital pair at position *j* of their joint list.

### **Type 3b/c/d blocking pairs**


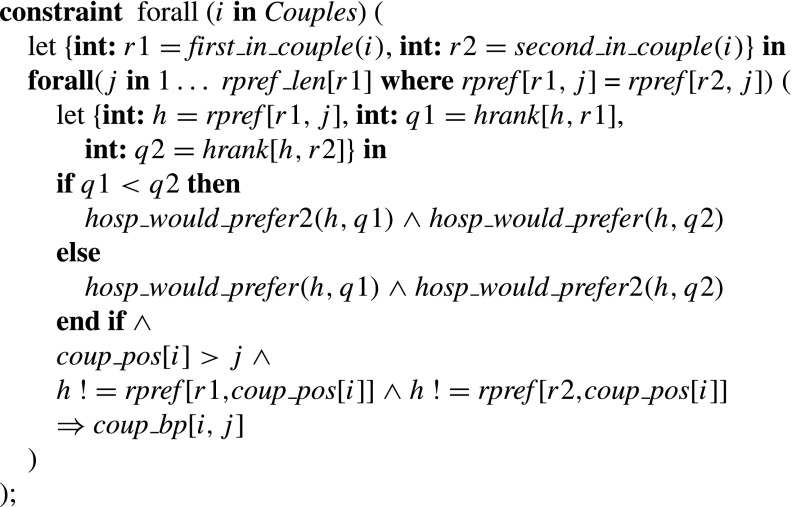


The *hosp*_*would*_*prefer*2 predicate for a hospital *h* and a position *q* on the preference list of *h* takes the value true if and only if *h* has fewer than *hosp*_*cap* [*h*]−1 assigned residents in positions strictly preferable to position *q* on its preference list. (Note the redundancy in this predicate: all we actually need is the first *s* *u* *m*(… )(… )<*hosp*_*cap* [*h*]−1 constraint; the *s* *u* *m*(… )(… )>1 constraint improves propagation.) 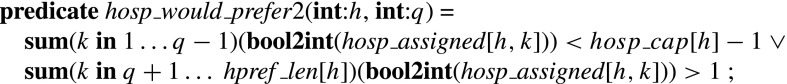


The constraint for Type 3b/c/d blocking pairs thus sets *coup*_*bp* [*i*,*j*] to true if and only if couple (*r*1,*r*2) are unassigned or prefer (*h*,*h*) to their joint assignment, whilst *h* either has two free posts (Type 3b), or *h* has one free post and prefers one of *r*1 or *r*2 to at least one of its assignees (Type 3c), or *h* is full and and prefers *r*1 to some assignee *rk*, and prefers *r*2 to at least one of its assignees apart from *rk* (Type 3d), where (*r*1,*r*2) is the *i*th couple and (*h*,*h*) is the hospital pair at position *j* of their joint list.

### Experiments

The CP model was solved using the lazy clause solver Chuffed [41] on the same machine that was used for the experiments on the IP model as reported in Section 4. All instances were allowed to run to completion. We present results on the runtime of the CP model both with and without presolving. The presolve step, when included, specifies in advance which set *S* of resident-hospital pairs will block the solution (in practice we try out values of *k*=0,1,2,… and generate all subsets *S* of size *k* until we reach a feasible solution) and then performs preference list deletions in the knowledge that the pairs in *S* will block. This allows large reductions in the model size, and works well because the number of blocking pairs admitted by a most-stable matching is generally very small, as we saw in Section 4. We did not use presolve with the IP model, but we note that it may be possible to solve the IP model more quickly by carrying out this step.

**Fig. 6:**
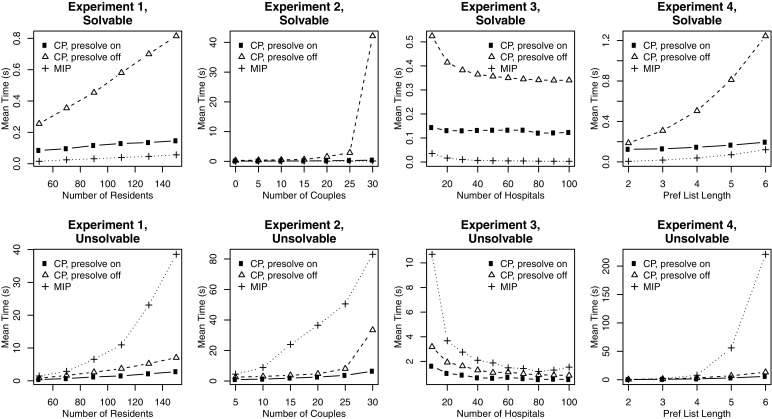
Comparison of run times using CP (with and without presolve) and MIP models

Figure 6 plots the mean run times for each of the four experiments for the IP model and for the CP models with and without presolving: each plot in the top row shows results for the solvable instances in one experiment, and each plot in the bottom row shows corresponding results for the unsolvable instances. Table 1 shows the actual mean and median runtimes for each model, taken over all 28,000 instances $\mathcal I$ across all four experiments, those instances from $\mathcal I$ that were solvable and those from $\mathcal I$ that were unsolvable.

**Table 1:** Summary of mean and median runtimes over all experiments (all timings are in seconds)

Instance type	Mean			Median		
	IP model	CP model		IP model	CP model	
		No presolve	Presolve		No presolve	Presolve
All	2.568	2.237	0.315	0.031	0.430	0.129
Solvable	0.034	1.839	0.143	0.016	0.402	0.127
Unsolvable	30.781	6.669	1.276	8.948	3.240	1.395

The CP model without presolve generally performs unfavourably for solvable instances. Here, the IP model is faster than the CP model with presolve; this is likely to be due to the fact that for such instances, the earlier IP model for hrc [7] is used instead of the more complex IP formulation for min bp hrc. For unsolvable instances, the CP model (with or without presolve) is faster than the IP model. This is likely to be due to the fact that the CP model for min bp hrc is more compact than its IP counterpart, involving fewer variables and constraints. Comparing total run time summed across all 28,000 instances, the CP model was 1.15 times faster than the CP model without presolve, and the CP model with presolve was 8.14 times faster than the IP model.

When solving the CP model, the distribution of runtimes for the case without presolve had a very long right tail; 14 of the 28,000 instances accounted for over half of the total run time. The longest-running instance took 17,617 seconds, and surprisingly this was a solvable instance (generated for Experiment 2). For this reason, Table 1 shows median run times as well as mean run times; from this we can see that the median runtime for the IP model is lower than that for the CP models for all instances and for solvable instances. However for unsolvable instances, the median runtime for CP without presolve is 2.762 times faster than the median runtime for IP, and this factor increases to 6.414 for CP with presolve.

## Concluding remarks

In this paper we have presented complexity and approximability results for min bp hrc, showing that the problem is NP-hard and very difficult to approximate even in highly restricted cases. We have then presented IP and CP models, together with empirical analyses of both models applied to randomly-generated hrc instances. Our main finding is that most-stable matchings admit a very small number of blocking pairs (in most cases at most 1, but never more than 2) on the instances we generated. We also showed that on average the CP model is faster than the IP model, with the performance of the CP model being enhanced if presolving was carried out. As far as future work is concerned, it would be interesting to determine the effect of presolving on the IP model, and more generally, to investigate further methods to enable the models to be scaled up to larger instances, such as column generation in the case of the IP model, and variable / value ordering heuristics in the case of the CP model.

## Electronic supplementary material

Below is the link to the electronic supplementary material.

(PDF 424 KB)

## Footnotes

1 All generated instances can be obtained from 10.5525/gla.researchdata.303.

## References

[1] Abraham, D.J., Biró, P., & Manlove, D.F (2006). “Almost stable” matchings in the Roommates problem. In *Proceedings of WAOA ’05: the 3rd Workshop on Approximation and Online Algorithms, volume 3879 of Lecture Notes in Computer Science* (pp. 1–14). Springer.

[2] Aldershof B, Carducci OM (1996). Stable matching with couples. Discrete Applied Mathematics.

[3] Balinski M, Sönmez T (1999). A tale of two mechanisms: student placement. Journal of Economic Theory.

[4] Biró, P. (2008). Student admissions in Hungary as Gale and Shapley envisaged. Technical Report TR-2008-291. University of Glasgow, Department of Computing Science.

[5] Biró, P., Irving, R.W., & Schlotter, I. (2011). Stable matching with couples: an empirical study. *ACM Journal of Experimental Algorithmics*, 16. Section 1, article 2.

[6] Biró, P., & Klijn, F. (2013). Matching with couples: a multidisciplinary survey. *International Game Theory Review*, *15*(2). article number 1340008.

[7] Biró, P., Manlove, D.F., & McBride, I. (2014). The Hospitals / Residents problem with couples: complexity and integer programming models. In *Proceedings of SEA '14: the 8th Symposium on Experimental Algorithms, volume 8504 of Lecture Notes in Computer Science* (pp. 10–21). Springer.

[8] Biró P, Manlove DF, McDermid EJ (2012). “Almost-stable” matchings in the Roommates problem with bounded preference lists. Theoretical Computer Science.

[9] Biró P, Manlove DF, Mittal S (2010). Size versus stability in the marriage problem. Theoretical Computer Science.

[10] Drummond, J., Perrault, A., & Bacchus, F. (2015). SAT is an effective and complete method for solving stable matching problems with couples. In *Proceedings of IJCAI ’15: the Twenty-fourth International Joint Conference on Artificial Intelligence* (pp. 518–525). AAAI Press.

[11] Eriksson K, Häggström O (2008). Instability of matchings in decentralized markets with various preference structures. International Journal of Game Theory.

[12] Floréen P, Kaski P, Polishchuk V, Suomela J (2010). Almost stable matchings by truncating the Gale-Shapley algorithm. Algorithmica.

[13] Gale D, Shapley LS (1962). College admissions and the stability of marriage. American Mathematical Monthly.

[14] Gent, I.P., Irving, R.W., Manlove, D.F., Prosser, P., & Smith, B.M. (2001). A constraint programming approach to the stable marriage problem. In *Proceedings of CP ’01: the 7th International Conference on Principles and Practice of Constraint Programming, volume 2239 of Lecture Notes in Computer Science* (pp. 225–239). Springer.

[15] Gusfield, D., & McDermid, R.W (1989). *The stable marriage problem: structure and algorithms*. MIT Press.

[16] Hamada K, Iwama K, Miyazaki S (2009). An improved approximation lower bound for finding almost stable stable maximum matchings. Information Processing Letters.

[17] Hamada K, Iwama K, Miyazaki S (2016). The hospitals/residents problem with lower quotas. Algorithmica.

[18] Hinder, O. (2015). The stable matching linear program and an approximate rural hospital theorem with couples. In *Proceedings of WINE '15: the 11th Conference on Web and Internet Economics, volume 9470 of Lecture Notes in Computer Science* (pp. 433). Springer. Full version available from http://stanford.edu/ohinder/stability-and-lp/working-paper.pdf.

[19] Irving, R.W. (1998). Matching medical students to pairs of hospitals: a new variation on a well-known theme. In *Proceedings of ESA ’98: the 6th Annual European Symposium on Algorithms, volume 1461 of Lecture Notes in Computer Science* (pp. 381–392). Springer.

[20] Manlove, D.F (2013). *Algorithmics of Matching Under Preferences*. World Scientific.

[21] Manlove, D.F., O’Malley, G., Prosser, P., & Unsworth, C. (2007). A constraint programming approach to the hospitals / residents problem. In *Proceedings of CP-AI-OR 2007: the 4th International Conference on Integration of AI and OR Techniques in Constraint Programming for Combinatorial Optimization, volume 4510 of Lecture Notes in Computer Science* (pp. 155–170). Springer.

[22] Marx D, Schlotter I (2011). Stable assignment with couples: parameterized complexity and local search. Discrete Optimization.

[23] McBride, I. (2015). Complexity and integer programming models for generalisations of the hospitals / residents problem. PhD thesis, School of Computing Science, University of Glasgow.

[24] McDermid EJ, Manlove DF (2010). Keeping partners together: algorithmic results for the hospitals / residents problem with couples. Journal of Combinatorial Optimization.

[25] Ng, C., & Hirschberg, D.S. (1988). Complexity of the stable marriage and stable roommate problem in three dimensions. Technical Report UCI-ICS 88–28, Department of Information and Computing Science, University of California, Irvine.

[26] Nguyen, T., & Vohra, R. (2015). Near feasible stable matchings. In *Proceedings of EC ’15: the Sixteenth ACM Conference on Economics and Computation* (pp 41–42). ACM.

[27] Perrault, A., Drummond, J., & Bacchus, F. (2015). Exploring strategy-proofness, uniqueness, and Pareto-optimality for the stable matching problem with couples. Technical Report arxiv:1505.03463, Computing Research Repository, Cornell University Library. Available from http://arxiv.org/abs/1505.03463.

[28] Robards, P.A. (2001). Applying two-sided matching processes to the United States Navy enlisted assignment process. Master’s thesis, Naval Postgraduate School, Monterey, CA.

[29] Romero-Medina A (1998). Implementation of stable solutions in a restricted matching market. Review of Economic Design.

[30] Ronn E (1990). NP-complete stable matching problems. Journal of Algorithms.

[31] Roth AE (1984). The evolution of the labor market for medical interns and residents: a case study in game theory. Journal of Political Economy.

[32] Roth AE (1990). New physicians: a natural experiment in market organization. Science.

[33] Roth E (1991). A natural experiment in the organization of entry level labor markets: regional markets for new physicians and surgeons in the UK. American Economic Review.

[34] Short, M.M. (2000). Analysis of the current navy enlisted detailing process. Master’s thesis, Naval Postgraduate School. Monterey, CA.

[35] Soldner, M. (2014). Optimization and measurement in humanitarian operations: Addressing practical needs. PhD thesis, Georgia Institute of Technology.

[36] Yang, W., Giampapa, J.A., & Sycara, K. (2003). Two-sided matching for the U.S. Navy Detailing Process with market complication. Technical Report CMU-RI-TR-03-49. Robotics Institute, Carnegie-Mellon University.

[37] Canadian Resident Matching Service website. http://www.carms.ca.

[38] Central Applications Office Ireland website. http://www.cao.ie.

[39] Japan Resident Matching Program website. http://www.jrmp.jp.

[40] Matching in Practice website. http://www.matching-in-practice.eu/.

[41] Higher Education Allocation in Ireland - Matching in Practice Website. http://www.matching-in-practice.eu/higher-education-in-ireland.

[42] National Resident Matching Program website. http://www.nrmp.org.

