# Supplementary material for: “Almost-stable” matchings in the Hospitals / Residents problem with Couples
Source: Constraints. 2016 Aug 11;22(1):50–72. doi: 10.1007/s10601-016-9249-7 (PMC7115081; doi:10.1007/s10601-016-9249-7)
Supplement: Supplementary file 1 — (PDF 424 KB) [file 10601_2016_9249_MOESM1_ESM.pdf]

Online supplement to:

“Almost-stable” matchings in the Hospitals / Residents problem with Couples

A Comparison of stability definitions

In this section we compare our stability definition for HRC given by Definition 1 with the definition adopted by Drummond et al. [43]. Suppose that I is an instance of HRC and M is a matching in I. Let $R' \subseteq R$. For a given hospital h_j, Drummond et al. defined $Ch_j(R')$ to be the set of residents that h_j would select from R'. That is, $Ch_j(R')$ is the maximal subset of R' such that, for all $r_i \in Ch_j(R')$, h_j finds r_i acceptable, h_j prefers r_i to any $r_k \in R' \backslash Ch_j(R')$ and $|Ch(R')| \leq c_j$. Then Drummond et al. defined the predicate $\mathsf{willAccept}(h_j, R', M)$ to be true if and only if $R' \subseteq Ch_j(M(h_j) \cup R')$.

Now suppose that (r_i, r_j) is a couple in I who prefer the hospital pair (h_k, h_k) to their assigned hospital pair $(M(r_i), M(r_j))$, where h_k is full in M. According to Condition 3(b) of the stability definition of Drummond et al. [43], h_k will participate in a blocking pair with (r_i, r_j) if and only if $\mathsf{willAccept}(h_k, \{r_i, r_j\}, M)$. According to Condition 3(d) of Definition 1, h_k will participate in a blocking pair with (r_i, r_j) if and only if h_k prefers r_i to some $r_s \in M(h_k)$, and h_k prefers r_j to some $r_t \in M(h_k) \backslash \{r_s\}$. Our Condition 3(d) is thus weaker than Condition 3(b) of Drummond et al. [43], meaning that our stability definition is stricter.

To illustrate the difference, consider the HRC instance I shown in Figure 7. Here h_1 has capacity 2, whilst each of h_2 and h_3 has capacity 1.

Residents	Hospitals
$(r_1, r_2) : (h_1, h_1) \quad (h_2, h_3)$	$h_1 : r_1 \quad r_3 \quad r_2 \quad r_4$
$r_3 : h_1$	$h_2 : r_1$
$r_4 : h_1$	$h_3 : r_1$

Fig. 7. An instance of HRC.

Let M be the matching $\{(r_1, h_2), (r_2, h_3), (r_3, h_1), (r_4, h_1)\}$. Then (r_1, r_2) forms a blocking pair of M with the hospital pair (h_1, h_1) according to the stability definition given in Definition 1, but this does not happen with respect to the stability definition of Drummond et al. [43]. In the latter case, $Ch_j(M(h_1) \cup \{r_1, r_2\}) = Ch_j(\{r_1, r_2, r_3, r_4\}) = \{r_1, r_3\} \not\supseteq \{r_1, r_2\}$. We would argue that (r_1, r_2) should form a blocking pair with (h_1, h_1), because h_1 unequivocally improves by rejecting $\{r_3, r_4\}$ and taking on $\{r_1, r_2\}$ instead.

B Inapproximability result for $(\infty, 1, \infty)$-MIN BP HRC

We now establish that the problem of deciding whether an instance of $(\infty, 1, \infty)$-HRC admits a stable matching is NP-complete.

Theorem 8 *Given an instance of* $(\infty, 1, \infty)$-HRC*, the problem of deciding whether there exists a stable matching is NP-complete. The result holds even if each hospital has capacity 1.*

Proof. The proof of this result uses a reduction from a restricted version of the vertex cover problem. More specifically, let VC3 denote the problem of deciding, given a cubic graph G and an integer K, whether G contains a vertex cover of size at most K. This problem is NP-complete [44, 45].

The problem of deciding whether there exists a stable matching in an instance of $(\infty, 1, \infty)$-HRC is clearly in NP, as a given assignment may be verified to be a stable matching in polynomial time. To show NP-hardness, let $\langle G, K \rangle$ be an instance of VC3, where $G = (V, E)$, $E = \{e_1, \ldots, e_m\}$ and $V = \{v_1, \ldots, v_n\}$. For each i $(1 \leq i \leq n)$, suppose that v_i is incident to edges e_{j_1}, e_{j_2} and e_{j_3} in G, where without loss of generality $j_1 < j_2 < j_3$. Define $e_{i,s} = e_{j_s}$ $(1 \leq s \leq 3)$. Similarly, for each j $(1 \leq j \leq m)$, suppose that $e_j = \{v_{i_1}, v_{i_2}\}$, where without loss of generality $i_1 < i_2$. Define $v_{j,r} = v_{i_r}$ $(1 \leq r \leq 2)$.

We form an instance I of $(\infty, 1, \infty)$-HRC as follows. The set of residents in I is $A \cup B \cup F \cup R \cup X \cup Y$ where $A = \{a_t : 1 \leq t \leq K\}$, $B = \{b_t : 1 \leq t \leq n - K\}$, $F = \bigcup_{t=1}^{K} F_t$, where $F_t = \{f_t^s : 1 \leq s \leq 6\}$, $R = \bigcup_{j=1}^{m} R_j$, where $R_j = \{r_j^s : 1 \leq s \leq 4\}$, $X = \{x_i : 1 \leq i \leq n\}$ and $Y = \bigcup_{t=1}^{n-K} Y_t$, where $Y_t = \{y_t^s : 1 \leq s \leq 6\}$.

The set of hospitals in I is $G \cup H \cup P \cup Q \cup Z$, where $G = \bigcup_{t=1}^{K} G_t$, where $G_t = \{g_t^r : 1 \leq r \leq 3\}$ $(1 \leq t \leq K)$, $H = \bigcup_{j=1}^{m} H_j$, $H_j = \{h_j^s : 1 \leq s \leq 2\}$, $P = \{p_t : 1 \leq t \leq K\}$, $Q = \{q_t : 1 \leq t \leq n - K\}$ and $Z = \bigcup_{t=1}^{n-K} Z_t$, where $Z_t = \{z_t^r : 1 \leq r \leq 3\}$ and each hospital has capacity 1. The preference lists of the resident couples, single residents and hospitals in I are shown in Figure 8.

In the preference list of a resident x_i $(1 \leq i \leq n)$ the symbol $h^s(x_i)$ $(1 \leq s \leq 3)$ denotes the hospital h_j^r $(1 \leq r \leq 2)$ such that $e_j = e_{i,s}$ and $v_i = v_{j,r}$. Similarly, in the preference list of a hospital h_j^r $(1 \leq j \leq m, 1 \leq r \leq 2)$ the symbol $x(h_j^r)$ denotes the resident x_i such that $v_i = v_{j,r}$.

We claim that G contains a vertex cover of size at most K if and only if I admits a stable matching. Let C be a vertex cover in G such that $|C| \leq K$. Without loss of generality we may assume that $|C| = K$ for if otherwise a sufficient number of vertices can be added to C without violating the vertex cover condition.

We show how to define a matching M in I as follows. Let $C = \{v_{r_1}, v_{r_2}, \ldots, v_{r_K}\}$ where without loss of generality $r_1 < r_2 < \ldots < r_K$. Further let $V \setminus C = \{v_{s_1}, v_{s_2}, \ldots, v_{s_{n-K}}\}$ where without loss of generality $s_1 < s_2 < \ldots < s_{n-k}$. For each vertex $v_{r_i} \in C$ add the pairs $\{(x_{r_i}, p_i), (a_i, g_i), (f_i^3, g_i^2), (f_i^4, g_i^3)\}$ for $1 \leq i \leq K$ to M. For each vertex $v_{s_i} \in V \setminus C$ add $\{(x_{s_i}, q_i), (b_i, z_i), (y_i^3, z_i^2), (y_i^4, z_i^3)\}$ for $1 \leq i \leq n - K$ to M.

Residents' Preferences

$(r_j^1, r_j^2) : (h_j^1, h_j^2)$	$(1 \leq j \leq m)$
$(r_j^3, r_j^4) : (h_j^1, h_j^2)$	$(1 \leq j \leq m)$
$(f_t^1, f_t^2) : (g_t^1, g_t^2)$	$(1 \leq t \leq K)$
$(f_t^3, f_t^4) : (g_t^2, g_t^3)$	$(1 \leq t \leq K)$
$(f_t^5, f_t^6) : (g_t^3, g_t^1)$	$(1 \leq t \leq K)$
$(y_t^1, y_t^2) : (z_t^1, z_t^2)$	$(1 \leq t \leq n-K)$
$(y_t^3, y_t^4) : (z_t^2, z_t^3)$	$(1 \leq t \leq n-K)$
$(y_t^5, y_t^6) : (z_t^3, z_t^1)$	$(1 \leq t \leq n-K)$
$a_t : p_t \;\; g_t$	$(1 \leq t \leq K)$
$b_t : q_t \;\; z_t$	$(1 \leq t \leq n-K)$
$x_i : p_1 \;\; p_2 \;\; \ldots \;\; p_K \;\; h^1(x_i) \;\; h^2(x_i) \;\; h^3(x_i) \;\; q_1 \;\; q_2 \;\; \ldots \;\; q_{n-K}$	$(1 \leq i \leq n)$

Hospitals' Preferences

$g_t^1 : a_t \;\; f_t^1 \;\; f_t^6$	$(1 \leq t \leq K)$
$g_t^2 : f_t^3 \;\; f_t^2$	$(1 \leq t \leq K)$
$g_t^3 : f_t^5 \;\; f_t^4$	$(1 \leq t \leq K)$
$h_j^1 : r_j^1 \;\; x(h_j^1) \;\; r_j^3$	$(1 \leq j \leq m)$
$h_j^2 : r_j^4 \;\; x(h_j^2) \;\; r_j^2$	$(1 \leq j \leq m)$
$p_t : x_1 \;\; x_2 \;\; \ldots \;\; x_n \;\; a_t$	$(1 \leq t \leq K)$
$q_t : x_1 \;\; x_2 \;\; \ldots \;\; x_n \;\; b_t$	$(1 \leq t \leq n-K)$
$z_t^1 : b_t \;\; y_t^1 \;\; y_t^6$	$(1 \leq t \leq n-K)$
$z_t^2 : y_t^3 \;\; y_t^2$	$(1 \leq t \leq n-K)$
$z_t^3 : y_t^5 \;\; y_t^4$	$(1 \leq t \leq n-K)$

Fig. 8. Preference lists in I, the constructed instance of $(\infty, 1, \infty)$-HRC.

For each edge $e_j \in E$ at least one of $v_{j,1}$ or $v_{j,2}$ must be in C. If $v_{j,1} \in C$ add the pairs $\{(r_j^3, h_j^1), (r_j^4, h_j^2)\}$ to M. Otherwise $v_{j,2} \in C$ so add the pairs $\{(r_j^1, h_j^1), (r_j^2, h_j^2)\}$ to M.

We now show that M is a stable matching in I. Firstly, we show that no hospital $h_j^r \in H$ $(1 \leq j \leq m, 1 \leq r \leq 2)$ can form part of a blocking pair of M. Assume a hospital $h_j^r \in H$ is part of a blocking pair of M for some j $(1 \leq j \leq m)$ and r $(1 \leq r \leq 2)$. Now, since C is a vertex cover in G, an arbitrary edge $e_j \in E$ must covered by either $v_{j,1}$ or $v_{j,2}$ or both. Assume firstly that $v_{j,1} \in C$. Then by construction $(x_{r_t}, p_t) \in M$ and $\{(r_j^3, h_j^1), (r_j^4, h_j^2)\} \subseteq M$ where $v_{j,1} = v_{r_t}$. Assume $(x(h_j^1), h_j^1)$ blocks M for some j $(1 \leq j \leq m)$. Since $v_{j,1} \in C$ and thus $M(x(h_j^1)) \in P$, $x(h_j^1)$ prefers $M(x(h_j^1))$ to h_j^1, a contradiction. Now assume that $((r_j^1, r_j^2), (h_j^1, h_j^2))$ blocks M. However, h_j^2 prefers $M(h_j^2) = r_j^4$ to r_j^2, a contradiction.

Now assume $v_{j,1} \notin C$. Then $v_{j,2} \in C$ and by construction $(x_{r_{t'}}, p_{t'}) \in M$ and $\{(r_j^1, h_j^1),\ (r_j^2, h_j^2)\} \subset M$ where $v_{j,2} = v_{r_{t'}}$. Assume $(x(h_j^2), h_j^2)$ blocks M for some j $(1 \leq j \leq m)$. Since $v_{j,2} \in C$ and thus $M(x(h_j^2)) \in P$, $x(h_j^2)$ prefers $M(x(h_j^2))$ to h_j^2, a contradiction. Now assume $((r_j^3, r_j^4), (h_j^1, h_j^2))$ blocks M. However, h_j^1 prefers $M(h_j^1) = r_j^1$ to r_j^3, a contradiction. Thus, no $h_j^r \in H$ $(1 \leq j \leq m, 1 \leq r \leq 2)$ can form part of a blocking pair of M.

We now show that no hospital in $P \bigcup Q$ can be involved in a blocking pair of M. By construction $M(p_t) \in X$ for all t $(1 \leq t \leq K)$. Assume some pair (x_{k_1}, p_{l_1}) blocks M. Let $M(x_{k_1}) = p_{l_2}$ and $M(p_{l_1}) = x_{k_2}$. Since (x_{k_1}, p_{l_1}) blocks M then $l_1 < 1_2$ and $k_1 < k_1$ in contradiction to the construction of M. A similar argument shows that no hospital in Q may be involved in a blocking pair of M and thus we have that no hospital in $P \bigcup Q$ may be involved in a blocking pair of M.

We now show that no hospital in $G \bigcup Z$ can be involved in a blocking pair of M. Firstly, assume a hospital $g_t^s \in H$ is part of a blocking pair of M for some t $(1 \leq t \leq K)$ and s $(1 \leq s \leq 3)$. Clearly, since g_t^1 and g_t^2 are both assigned their first preference they cannot form part of a blocking pair for M. Hospital g_t^3 prefers f_t^5 to $M(g_t^3) = f_t^4$. However, f_t^5 is a member of the couple (f_t^5, f_t^6) that expresses a joint preference for the pair (g_t^3, g_t^1) and g_t^1 prefers $M(g_t^1) = a_t$ to f_t^6, a contradiction. Thus, no hospital $g_t^s \in H$ $(1 \leq t \leq K, 1 \leq s \leq 3)$ can form part of a blocking pair of M. A similar argument may be used to show that no $z_t^s \in H$ $(1 \leq t \leq n-K, 1 \leq s \leq 3)$ can form part of a blocking pair of M and thus we have that no hospital in $G \bigcup Z$ can be involved in a blocking pair of M.

We now have that no hospital in I may be part of a blocking pair of M and thus M must be stable.

Conversely, let M be a stable matching in I. We first show that the stability of M implies that $M(x_i) \in P \bigcup Q$ for all i $(1 \leq i \leq n)$. Observe that if $(a_t, g_t^1) \notin M$ for t $(1 \leq t \leq K)$ then no stable assignment is possible amongst the agents in $F_t \bigcup G_t$ as shown in Lemma 13. However, if $\{(a_t, g_t^1),\ (f_t^3, g_t^2),\ (f_t^4, g_t^3)\} \subseteq M$ then no blocking pair exists in $F_t \bigcup G_t$. It follows that if $(a_t, g_t^1) \in M$ then (a_t, p_t) blocks M unless $M(p_t) \in X$. A similar argument shows that $M(q_t) \in X$

for all t ($1 \leq t \leq n-K$). Now, since $|X| = n$ and $|P \bigcup Q| = n$, clearly all $x \in X$ must be partnered with a member of $P \bigcup Q$ and moreover, $M(x_i) \notin H$ in any stable matching in I.

Next we show that the stability of M implies that h_j^1 and h_j^2 are fully subscribed in M for all j ($1 \leq j \leq m$). Let j ($1 \leq j \leq m$) be given. Assume that both h_j^1 and h_j^2 are undersubscribed in M. Since $M(x(h_j^r)) \neq h_j^r$ for all j, r ($1 \leq j \leq m, 1 \leq r \leq 2$), $((r_j^1, r_j^2), (h_j^1, h_j^2))$ blocks M, a contradiction. Thus either $\{(r_j^1, h_j^1), (r_j^2, h_j^2))\} \subseteq M$ or $\{(r_j^3, h_j^1), (r_j^4, h_j^2))\} \subseteq M$ in any stable matching in I. If $\{(r_j^1, h_j^1), (r_j^2, h_j^2))\} \subseteq M$ then $((r_j^3, r_j^4), (h_j^1, h_j^2))$ does not block M. Similarly, if $\{(r_j^3, h_j^1), (r_j^4, h_j^2))\} \subseteq M$ then $((r_j^1, r_j^2), (h_j^1, h_j^2))$ does not block M. Thus we have that h_j^1 and h_j^2 are fully subscribed in M for all j ($1 \leq j \leq m$). Moreover, we have that all hospitals must be fully subscribed in any stable matching M in I.

Define a set of vertices C in G as follows. For each i ($1 \leq i \leq n$) if $M(x_i) \in P$, add v_i to C. Since M is a stable matching and $|P| = K$, this process selects exactly K of the n vertices in V and thus $|C| = K$. We now show that C represents a vertex cover in G. Consider an arbitrary edge $e_j \in E$. Assume that both $v_{j,1} \notin C$ and $v_{j,2} \notin C$ and hence that C is not a vertex cover in G. Then $M(x_{j,1}) \in Q$ and $M(x_{j,2}) \in Q$. As M is stable and thus hospital complete, either $\{(r_j^1, h_j^1), (r_j^2, h_j^2)\} \subset M$ or $\{(r_j^3, h_j^1), (r_j^4, h_j^2)\} \subset M$. If $\{(r_j^1, h_j^1), (r_j^2, h_j^2)\} \subset M$ then $(x_{j,2}, h_j^2)$ blocks M, a contradiction. If $\{(r_j^3, h_j^1), (r_j^4, h_j^2)\} \subset M$ then $(x_{j,1}, h_j^1)$ blocks M, a contradiction. Hence C represents a vertex cover in G of size K and the theorem is proven. □

Corollary 9 $(\infty, 1, \infty)$-MIN BP HRC *is NP-hard and not approximable within a factor of* $n_1^{1-\varepsilon}$, *for any* $\varepsilon > 0$, *unless P=NP, where* n_1 *is the number of residents in a given instance. The result holds even if each hospital has capacity 1.*

Proof. The proof of this result is analogous to the proof of Theorem 2 in [46], which establishes the same result for $(0, 2, 2)$-MIN BP HRC, using the NP-completeness of the problem of deciding whether a stable matching exists in a given instance of $(0, 2, 2)$-HRC as a starting point. The restrictions on the preference list lengths are not used in the gap-introducing reduction that proves the inapproximability result, hence the same reduction can be used to demonstrate the inapproximability of $(\infty, 1, \infty)$-MIN BP HRC. □

C Efficiently solvable variants of MIN BP HRC

C.1 Fixed assignments in HRC

In an instance I of HRC some agents may rank one another highly in their preference lists, leading to the outcome that they must be assigned to one another in any stable matching in I. We describe these agents as *fixed assignments*, using the following lemma to define this concept formally and to show that fixed assignments must belong to any stable matching in I.

Lemma 10 *Let I be an arbitrary instance of* HRC.

(i) If a single resident r_i has a hospital h_j in first place on his preference list and r_i is within the first c_j places on h_j's preference list then (r_i, h_j) must belong to any stable matching in I.
(ii) If a couple (r_i, r_j) has a hospital pair (h_p, h_q) in first place on its joint preference list and r_i is within the first c_p places on h_p's preference list and also r_j is within the first c_q places on h_q's preference list then (r_i, r_j) must be jointly assigned to (h_p, h_q) in any stable matching in I.

Any pair consisting of a single resident and a single hospital satisfying Case (i), or consisting of a couple and hospital pair satisfying Case (ii), is called a fixed assignment *in I.*

Proof. The proof of the Lemma follows immediately from the fact that any matching M in which r_i is not assigned to h_j will be blocked by (r_i, h_j), and similarly, any matching M in which (r_i, r_j) and (h_p, h_q) are not assigned to each other will be blocked by (r_i, r_j) with (h_p, h_q). □

Suppose that a matching M is constructed solely by matching agents who are involved in fixed assignments. As a consequence, suppose some pair (r_i, h_j) is added to M. Clearly no other hospital may be assigned in M to r_i, and hence r_i can be deleted from the preference list of each other hospital in which he appears. Moreover, in the event that h_j becomes fully subscribed by accepting r_i as an assignee, h_j can be deleted from the preference list of each resident other than r_i in which it appears. We say that we *satisfy* a fixed assignment if we match together in M the agents involved, and then carry out the corresponding deletions as described above. Note that making these deletions may expose another fixed assignment in the resulting reduced instance of HRC, which can then also be satisfied in M. If we continue satisfying fixed assignments until no more fixed assignments are exposed then we say all fixed assignments have been *iteratively satisfied* in M. This idea is used in the proof of the following proposition, to show that a stable solution can be found in an instance of $(\infty, \infty, 1)$-HRC in polynomial time.

Proposition 11 *An instance I of $(\infty, \infty, 1)$-HRC admits exactly one stable matching, which can be found in polynomial time.*

Proof. Consider an arbitrary single resident r_i in I. Let the hospital in first place on resident r's preference list be h_j. Since r_i must be in first place in h_j's preference list (as it is the only preference expressed by h_j), the pair (r_i, h_j) represents a fixed assignment in I. Thus, any single resident in I must be part of exactly one fixed assignment in I and this may be satisfied by assigning each single resident to the hospital in first place on his preference list.

Now, consider an arbitrary couple (r_i, r_j) in I. Let the hospital pair (h_p, h_q) be in first place on couple (r_i, r_j)'s joint preference list. Clearly, since r_i (respectively r_j) is in first place on h_p's (respectively h_q's) preference list, (r_i, r_j)

with (h_p, h_q) represents a fixed assignment in I. Thus, any resident couple in I must be part of exactly one fixed assignment in I and this may be satisfied by assigning each couple to the hospital pair in first place on their joint preference list.

Hence, the fixed assignments involving both the single residents and the couples in I may be satisfied iteratively in time linear in the number of residents in I, leading to a matching M that is clearly stable in I, and which is the only stable matching in I. □

C.2 (2, 1, 2)-MIN BP HRC is efficiently solvable

Let I be an instance of (2, 1, 2)-HRC, and assume that M_0 is a matching in I in which all fixed assignments have been iteratively satisfied, and assume that the corresponding deletions have been made from the preference lists in I. In Lemma 12 below, we use the absence of fixed assignments in I to infer that I must be constructed from the union of a finite number of disjoint discrete sub-instances of (2, 1, 2)-HRC and further that each disjoint sub-instance I' of I must be of the form shown in Figure 9. Let I' be one of these disjoint sub-instances of I. We prove in Lemma 13 that the number of couples involved in I' determines whether I' admits a stable matching: indeed, I' admits a stable matching if and only if the number of couples in I' is even.

Lemma 12 *An arbitrary instance of* (2, 1, 2)-HRC *involving at least one couple and in which all fixed assignments have been iteratively satisfied must be constructed from sub-instances of the form shown in Figure 9 in which all of the hospitals have capacity 1.*

Proof. Let I be an arbitrary instance of (2, 1, 2)-HRC in which all fixed assignments have been iteratively satisfied. Observe that if a couple expresses a preference for a hospital pair (h_p, h_p) this would represent a fixed assignment, a contradiction. Thus, no couple may express such a preference in I. We now show how the absence of fixed assignments in I allows us to infer the preference lists for all of the agents involved in I.

Let $(r^1_{c_1}, r^2_{c_1})$ be a couple in I and further let $(h^0_{c_1}, h^1_{c_1})$ be the hospital pair for which $(r^1_{c_1}, r^2_{c_1})$ expresses a preference. Since all fixed assignments have been iteratively satisfied by construction, it cannot be the case that *both*:

(i) $h^0_{c_1}$ has capacity two or has $r^1_{c_1}$ in first place in its preference list *and*
(ii) $h^1_{c_1}$ has capacity two or has $r^2_{c_1}$ in first place in its preference list.

Without loss of generality, assume that $h^1_{c_1}$ has capacity one and does not have $r^2_{c_1}$ in first place in its preference list. Hence there exists some other resident r_x who is preferred by $h^1_{c_1}$. Clearly, this resident is either a member of a couple or is a single resident. We now consider both of these cases and show that the we must arrive at the same outcome in either case. In what follows n_k $(1 \leq k \leq n_N)$ represents the number of single residents generated following couple c_k as the preference lists of the residents are inferred in the proof.

Residents	Hospitals
$(r_{c_1}^1, r_{c_1}^2) : (h_{c_1}^0, h_{c_1}^1)$	$h_{c_1}^0 : r_{c_1}^1 \quad r_{s_N}^{n_N}$
$r_{s_1}^1 : h_{c_1}^2 \quad h_{c_1}^1$	$h_{c_1}^1 : r_{s_1}^1 \quad r_{c_1}^2$
$r_{s_1}^2 : h_{c_1}^3 \quad h_{c_1}^2$	$h_{c_1}^2 : r_{s_1}^2 \quad r_{s_1}^1$
$\vdots$	$\vdots$
$r_{s_1}^{n_1} : h_{c_1}^{n_1+1} \quad h_{c_1}^{n_1}$	$h_{c_1}^{n_1} : r_{s_1}^{n_1} \quad r_{s_1}^{n_1-1}$
$(r_{c_2}^1, r_{c_2}^2) : (h_{c_1}^{n_1+1}, h_{c_2}^1)$	$h_{c_1}^{n_1+1} : r_{c_2}^1 \quad r_{s_1}^{n_1}$
$r_{s_2}^1 : h_{c_2}^2 \quad h_{c_2}^1$	$h_{c_2}^1 : r_{s_2}^1 \quad r_{c_2}^2$
$r_{s_2}^2 : h_{c_2}^3 \quad h_{c_2}^2$	$h_{c_2}^2 : r_{s_2}^2 \quad r_{s_2}^1$
$\vdots$	$\vdots$
$r_{s_2}^{n_2} : h_{c_2}^{n_2+1} \quad h_{c_2}^{n_2}$	$h_{c_2}^{n_2} : r_{s_2}^{n_2} \quad r_{c_2}^{n_2-1}$
$(r_{c_3}^1, r_{c_3}^2) : (h_{c_2}^{n_2+1}, h_{c_3}^1)$	$h_{c_2}^{n_2+1} : r_{c_3}^1 \quad r_{s_2}^{n_2}$
$r_{s_3}^1 : h_{c_3}^2 \quad h_{c_3}^1$	$h_{c_3}^1 : r_{s_3}^1 \quad r_{c_3}^2$
$r_{s_3}^2 : h_{c_3}^3 \quad h_{c_3}^2$	$h_{c_3}^2 : r_{s_3}^2 \quad r_{s_3}^1$
$\vdots$	$\vdots$
$r_{s_{N-1}}^{n_{N-1}} : h_{c_{N-1}}^{n_{N-1}+1} \quad h_{c_{N-1}}^{n_{N-1}}$	$h_{c_{N-1}}^{n_{N-1}} : r_{s_{N-1}}^{n_{N-1}} \quad r_{c_{N-1}}^{n_{N-1}-1}$
$(r_{c_N}^1, r_{c_N}^2) : (h_{c_{N-1}}^{n_{N-1}+1}, h_{c_N}^1)$	$h_{c_{N-1}}^{n_{N-1}+1} : r_{c_N}^1 \quad r_{s_{N-1}}^{n_{N-1}}$
$r_{s_N}^1 : h_{c_N}^2 \quad h_{c_N}^1$	$h_{c_N}^1 : r_{s_N}^1 \quad r_{c_N}^2$
$r_{s_N}^2 : h_{c_N}^3 \quad h_{c_N}^2$	$h_{c_N}^2 : r_{s_N}^2 \quad r_{s_N}^1$
$\vdots$	$\vdots$
$r_{s_N}^{n_N} : h_{c_1}^0 \quad h_{c_N}^{n_N}$	$h_{c_N}^{n_N} : r_{s_N}^{n_N} \quad r_{s_N}^{n_N-1}$

Fig. 9. An instance of $(2, 1, 2)$-HRC containing an arbitrary number of couples and an arbitrary number of residents that has no unsatisfied fixed assignments.

Case (i): r_x is single and thus $n_1 > 0$. In this case let $r_x = r_{s_1}^1$. Since $r_{s_1}^1$ is in first place in the preference list of $h_{c_1}^1$, to prevent a fixed assignment, there must exist another hospital that is preferred by $r_{s_1}^1$; let this be $h_{c_1}^2$. If $h_{c_1}^2$ has capacity two then $(r_{s_1}^1, h_{c_1}^2)$ represents a fixed assignment, a contradiction. Hence, $h_{c_1}^2$ must have capacity one.

Now, since $r_{s_1}^1$ has $h_{c_1}^2$ in first place in its preference list, there must exist some other resident who is preferred by $h_{c_1}^2$. We consider first the case where each newly generated resident is single. Hence, let this new resident be a single resident, $r_{s_1}^2$. Since $r_{s_1}^2$ is in first place on the preference list of $h_{c_1}^2$ there must exist another hospital which is preferred by $r_{s_1}^2$; let this new hospital be $h_{c_1}^3$. Assume $h_{c_1}^3$ has capacity two. In that case $(r_{s_1}^1, h_{c_1}^2)$ represents a fixed assignment, a contradiction. Hence, $h_{c_1}^3$ must have capacity one.

We may continue constructing a sequence of distinct single residents and hospitals of capacity one, but as the number of single residents is finite, ultimately we must eventually arrive at a resident who is a member of a couple; let this

Residents

$(r^1_{c_1}, r^2_{c_1})$	:	$(h^0_{c_1}, h^1_{c_1})$	
$(r^1_{c_k}, r^2_{c_k})$	:	$(h^{n_{k-1}+1}_{c_{k-1}}, h^1_{c_k})$	$2 \leq k \leq N-1$
$(r^1_{c_N}, r^2_{c_N})$	:	$(h^{n_{N-1}+1}_{c_{N-1}}, h^1_{c_N})$	$n_N > 0$
$(r^1_{c_N}, r^2_{c_N})$	:	$(h^{n_{N-1}+1}_{c_{N-1}}, h^0_{c_1})$	$n_N = 0$
$r^a_{s_k}$	:	$h^{a+1}_{c_k} \quad h^a_{c_k}$	$1 \leq k \leq N, 1 \leq a \leq n_k, n_k > 0$

Hospitals

$h^0_{c_1}$	:	$r^1_{c_1} \quad r^{n_N}_{s_N}$	if $n_N > 0$
$h^0_{c_1}$	:	$r^1_{c_1} \quad r^2_{c_N}$	if $n_N = 0$
$h^1_{c_k}$	:	$r^1_{s_k} \quad r^2_{c_k}$	$1 \leq k \leq N$, if $n_k > 0$
$h^1_{c_k}$	:	$r^1_{c_{k+1}} \quad r^2_{c_k}$	$1 \leq k \leq N$, if $n_k = 0$
$h^a_{c_k}$	:	$r^a_{s_k} \quad r^{a-1}_{s_k}$	$1 \leq k \leq N$, $2 \leq a \leq n_k$, if $n_k > 0$
$h^{n_k+1}_{c_k}$	:	$r^1_{c_{k+1}} \quad r^{n_k}_{s_k}$	$1 \leq k \leq N-1$, if $n_k > 0$

Fig. 10. An exactly equivalent description of the instance shown in Figure 9

resident be $r^1_{c_2}$. Without loss of generality suppose that $r^1_{c_2}$ is the first member of the couple to which he belongs. Let $r^{n_1}_{s_1}$ be the final single resident constructed in the preceding sequence.

It follows that $r^{n_1}_{s_1}$ prefers some hospital $h^{n_1+1}_{c_1}$ of capacity one to $h^{n_1}_{c_1}$. If $h^{n_1+1}_{c_1} = h^0_{c_1}$ then I contains precisely one couple and the instance is of the form shown in Figure 9 where $N = 1$ and $n_1 > 0$. Otherwise $h^{n_1+1}_{c_1}$ is a new hospital of capacity one that prefers $r^1_{c_2}$ to $r^{n_1}_{s_1}$. Since $h^{n_1+1}_{c_1}$ has $r^1_{c_2}$ in first place on its preference list, it must be the case that $r^1_{c_2}$ expresses a joint preference as part of the couple $(r^1_{c_2}, r^2_{c_2})$ for a hospital pair involving $h^{n_1+1}_{c_1}$; let this pair be $(h^{n_1+1}_{c_1}, h^1_{c_2})$. Since $h^{n_1+1}_{c_1}$ has $r^1_{c_2}$ in first place on its preference list, $h^1_{c_2}$ must be of capacity one and prefer some other resident to $r^2_{c_2}$, otherwise $(r^1_{c_2}, r^2_{c_2})$ represents a fixed assignment with $(h^{n_1+1}_{c_1}, h^1_{c_2})$, a contradiction. Now, let this other resident be r_y.

Case (ii): r_x is a member of a couple and thus $n_1 = 0$. Let $r_x = r^1_{c_2}$. Then $h^1_{c_1}$ prefers $r^1_{c_2}$ to $r^2_{c_1}$. Assume that $r^1_{c_2}$ is part of a couple $(r^1_{c_2}, r^2_{c_2})$ and further assume that $(r^1_{c_2}, r^2_{c_2})$ finds $(h^1_{c_1}, h^1_{c_2})$ acceptable. If $h^1_{c_2} = h^0_{c_1}$ then I contains exactly two couples and is of the form shown in Figure 9 with $N = 2$ and $n_1 = n_2 = 0$. (In this case $h^0_{c_1}$ prefers $r^1_{c_1}$ to $r^2_{c_2}$.) Otherwise, $h^1_{c_2}$ is a new hospital which must be of capacity one, or $(r^1_{c_2}, r^2_{c_2})$ represents a fixed assignment with $(h^1_{c_1}, h^1_{c_2})$, and moreover $h^1_{c_2}$ must prefer some other resident to $r^2_{c_2}$; let this resident be r_y.

Thus in both cases we have that if $h^1_{c_2} \neq h^0_{c_1}$ then $h^1_{c_2}$ is of capacity one and prefers some resident r_y to $r^2_{c_2}$. Clearly, r_y is either a member of a couple or is a single resident. As before, we consider both of these cases and show that we must arrive at the same outcome in either case.
Case (i): r_y is single and thus $n_2 > 0$; In this case let $r_y = r^1_{s_2}$. Since $r^1_{s_2}$ is in first place on the preference list of $h^1_{c_2}$, it follows that $h^1_{c_2}$ cannot be in first place in the preference list of $r^1_{s_2}$. Hence, there must exist another hospital preferred by $r^1_{s_2}$; let this be $h^2_{c_2}$. Further, $h^2_{c_2}$ must be of capacity one and have a resident other than $r^1_{s_2}$ in first place in its preference list; let this resident be $r^2_{s_2}$. We consider first the case where each newly generated resident is single. Suppose $r^2_{s_2}$ is single. Since $r^2_{s_2}$ is in first place on the preference list of $h^2_{c_2}$ there must exist another hospital which is preferred by $r^2_{s_2}$; let this new hospital be $h^3_{c_2}$. Hospital $h^3_{c_2}$ must have capacity one, otherwise $(r^2_{s_2}, h^3_{c_2})$ would represent a fixed assignment.

We may continue generating a sequence of distinct single residents and hospitals of capacity one, but since the number of residents is finite, we must eventually arrive at a resident who is a member of a couple; let this resident be $r^1_{c_3}$. Without loss of generality suppose that $r^1_{c_3}$ is the first member of the couple to which he belongs. Let $r^{n_2}_{s_2}$ be the final single resident in the previously generated sequence. Then $r^{n_2}_{s_2}$ prefers some hospital $h^{n_2+1}_{c_2}$ to $h^{n_2}_{c_2}$ and $h^{n_2+1}_{c_2}$ must be of capacity one. If $h^{n_2+1}_{c_2} = h^0_{c_1}$ then I contains precisely two couples. Otherwise $h^{n_2+1}_{c_2}$ is a new hospital of capacity one and prefers $r^1_{c_3}$ to $r^{n_2}_{s_2}$.

Since $h^{n_2+1}_{c_2}$ has $r^1_{c_3}$ in first place on its preference list, it must be the case that $r^1_{c_3}$ expresses a joint preference as part of the couple $(r^1_{c_3}, r^2_{c_3})$ for a hospital pair involving $h^{n_2+1}_{c_2}$; let this pair be $(h^{n_2+1}_{c_2}, h^1_{c_3})$.

Since $h^1_{c_3}$ has $r^2_{c_3}$ in first place on its preference list, $h^2_{c_3}$ must be of capacity one and prefer some other resident to $r^2_{c_3}$; let this resident be r_z.
Case (ii): r_y is a member of a couple and thus $n_2 = 0$. Let $r_y = r^1_{c_2}$. Then $h^1_{c_2}$ prefers $r^1_{c_3}$ to $r^2_{c_2}$. Assume that $r^1_{c_3}$ is part of a couple $(r^1_{c_3}, r^2_{c_3})$ and further assume that $(r^1_{c_3}, r^2_{c_3})$ finds $(h^1_{c_2}, h^1_{c_3})$ acceptable. If $h^1_{c_3} = h^0_{c_1}$ then I contains three couples and is of the form shown in Figure 9 with $N = 3$ and $n_3 = 0$. (In this case $h^0_{c_1}$ prefers $r^1_{c_1}$ to $r^2_{c_3}$.) Otherwise, $h^1_{c_3}$ is a new hospital which must be of capacity one (or else $(r^1_{c_3}, r^2_{c_3})$ represents a fixed assignment with $(h^1_{c_2}, h^1_{c_3})$ and must prefer some resident to $r^2_{c_3}$; let this resident be r_z.

Now, in both cases we have that if $h^1_{c_3} \neq h^0_{c_1}$ then $h^1_{c_3}$ is of capacity one and prefers some resident r_z to $r^2_{c_3}$. As before, we may continue generating a sequence of distinct residents, couples and hospitals in this fashion, but since the number of residents and couples is finite, we must eventually reach some resident, either single or a member of a couple who must be in second place in $h^0_{c_1}$'s preference list and a complete instance of $(2,1,2)$-HRC is formed. Thus, the instance I must be of the form shown in Figure 9. □

Lemma 13 *An instance I of $(2,1,2)$-HRC of the form shown in Figure 9 admits a stable matching if and only if the number of couples involved in I is even.*

Proof. Let M be a stable matching in I. It is either the case that $(r^1_{c_1}, r^2_{c_1})$ is assigned in M or $(r^1_{c_1}, r^2_{c_1})$ is unassigned in M. We now consider each of these

cases and show that in either case if I contains an odd number of couples then I cannot admit a stable matching.

Case (i): Assume $(r^1_{c_1}, r^2_{c_1})$ is assigned in M and therefore $(r^1_{c_1}, h^0_{c_1}) \in M$. Clearly either $n_1 = 0$ or $n_1 > 0$. We now show that whether $n_1 = 0$ or $n_1 > 0$, if $(r^1_{c_1}, r^2_{c_1})$ is assigned in M then $(r^1_{c_2}, r^2_{c_2})$ is unassigned in M.

If $n_1 = 0$ and the instance contains exactly one couple, then $(r^1_{c_1}, r^2_{c_1})$ represents a fixed assignment with $(h^0_{c_1}, h^1_{c_1})$, a contradiction. Thus, I contains more than one couple. Let the second couple in I be $(r^1_{c_2}, r^2_{c_2})$ such that $h^1_{c_1}$ has $r^1_{c_2}$ in first place on its preference list. We now have that $(r^1_{c_2}, r^2_{c_2})$ expresses a preference for $(h^1_{c_1}, h^1_{c_2})$ and since $(r^2_{c_1}, h^1_{c_1}) \in M$, clearly $(r^1_{c_2}, r^2_{c_2})$ cannot be assigned in M.

If $n_1 > 0$ then $h^1_{c_1}$ has $r^1_{s_1}$ in first place on its preference list. Now, if $r^1_{s_1}$ is unassigned in M then $(r^1_{s_1}, h^1_{c_1})$ blocks M. Hence $r^1_{s_1}$ must be assigned in M and moreover $(r^1_{s_1}, h^2_{c_1}) \in M$. In similar fashion we may confirm that each $r^a_{s_1}$ $(1 \leq a < n_1)$ is assigned to the hospital $h^{a+1}_{c_1}$ in first place on its preference list.

Now consider, $r^{n_1}_{s_1}$. Again $r^{n_1}_{s_1}$ must be assigned to the hospital in first place in its preference list. If I contains exactly one couple then this hospital must be $h^0_{c_1}$ by Lemma 12. However, by assumption $(r^1_{c_1}, h^0_{c_1}) \in M$, a contradiction. Thus I must contain more than one couple. Now, let $h^{n_1+1}_{c_1}$ be the hospital in first place on $r^{n_1}_{s_1}$'s preference list. Since $(r^{n_1}_{s_1}, h^{n_1+1}_{c_1}) \in M$, clearly $(r^1_{c_2}, r^2_{c_2})$ cannot be assigned in M as the only pair they find acceptable is $(h^{n_1+1}_{c_1}, h^1_{c_2})$. Thus, we have that whether $n_1 = 0$ or $n_1 > 0$, if $(r^1_{c_1}, r^2_{c_1})$ is assigned in M then $(r^1_{c_2}, r^2_{c_2})$ is not assigned in M.

Now, either $n_2 = 0$ or $n_2 > 0$. We now show that whether $n_2 = 0$ or $n_2 > 0$, if $(r^1_{c_2}, r^2_{c_2})$ is unassigned in M then $(r^1_{c_3}, r^2_{c_3})$ must be assigned in M. If $n_2 = 0$ and the instance contains exactly two couples then $(r^1_{c_2}, r^2_{c_2})$ expresses a preference for either $(h^1_{c_1}, h^0_{c_1})$ if $n_1 = 0$ (or $(h^{n_1+1}_{c_1}, h^0_{c_1})$ if $n_1 > 0$) and $h^0_{c_1}$ has $r^2_{c_2}$ in second place on its preference list. In this case, the instance admits exactly two stable matchings of equal cardinality. If $n_2 = 0$ and the instance contains more than two couples then $(r^1_{c_3}, r^2_{c_3})$ expresses a preference for $(h^1_{c_2}, h^1_{c_3})$. Now assume, $h^1_{c_2}$ is unassigned in M. Then $(r^1_{c_2}, r^2_{c_2})$ blocks M with $(h^1_{c_1}, h^0_{c_1})$ if $n_1 = 0$ (or $(h^{n_1+1}_{c_1}, h^0_{c_1})$ if $n_1 > 0$), a contradiction. Thus we have that if $(r^1_{c_2}, r^2_{c_2})$ is not assigned in M then $(r^1_{c_3}, r^2_{c_3})$ must be assigned to $(h^1_{c_2}, h^1_{c_3})$ in M.

If $n_2 > 0$ then $h^1_{c_2}$ has $r^1_{s_2}$ in first place on its preference list. Now, if $r^1_{s_2}$ is not assigned in M then $(r^1_{s_2}, h^1_{c_2})$ blocks M, a contradiction. Hence $r^1_{s_2}$ must be assigned in M and moreover $(r^1_{s_2}, h^2_{c_2}) \in M$. In similar fashion we may confirm that each $r^a_{s_2}$ $(1 \leq a \leq n_2)$ must be assigned in M to the hospital $h^{a+1}_{s_2}$ in first place in its preference list.

Now consider, $r^{n_2}_{s_2}$. If the instance contains exactly two couples then the hospital in first place in the preference list of $r^{n_2}_{s_2}$ must be $h^0_{c_1}$ and the result follows. However, if the instance contains more than two couples then the hospital in first place in the preference list of $r^{n_2}_{s_2}$ must be a new hospital $h^{n_2+1}_{c_2}$. Now let the next couple be $(r^1_{c_3}, r^2_{c_3})$. Assume $(r^1_{c_3}, r^2_{c_3})$ is unassigned in M. Then $(r^{n_2}_{s_2}, h^{n_2+1}_{c_2})$ must block M, so $(r^1_{c_3}, r^2_{c_3})$ must be assigned to $(h^{n_2+1}_{c_2}, h^1_{c_3})$ in M.

Thus, whether $n_2 = 0$ or $n_2 > 0$, if $(r^1_{c_2}, r^2_{c_2})$ is unassigned in M then $(r^1_{c_3}, r^2_{c_3})$ must be assigned in M.

In similar fashion either $n_3 = 0$ or $n_3 > 0$. Again, we show that whether $n_3 = 0$ or $n_3 > 0$, if $(r^1_{c_3}, r^2_{c_3})$ is assigned in M then $(r^1_{c_4}, r^2_{c_4})$ is not assigned in M. If $n_3 = 0$ and the instance contains exactly three couples then $(r^1_{c_3}, r^2_{c_3})$ is assigned to $(h^1_{c_2}, h^0_{c_1})$ if $n_2 = 0$ (or $(h^{n_2+1}_{c_1}, h^0_{c_1}$ if $n_2 > 0)$ and $h^0_{c_1}$ has $r^2_{c_3}$ in second place on its preference list. However, by assumption $(r^2_{c_1}, h^0_{c_1}) \in M$, a contradiction. Thus, I contains more than three couples and $(r^1_{c_4}, r^2_{c_4})$ expresses a preference for $(h^1_{c_3}, h^1_{c_4})$ and since $(r^1_{c_3}, h^1_{c_3}) \in M$, $(r^1_{c_4}, r^2_{c_4})$ cannot be assigned in M.

If $n_3 > 0$ then $h^1_{c_3}$ has $r^1_{s_3}$ in first place on its preference list. Now, if $r^1_{s_3}$ is not assigned in M then $(r^1_{s_3}, h^1_{c_3})$ blocks M, a contradiction. Hence $r^1_{s_3}$ must be assigned in M and moreover $(r^1_{s_3}, h^2_{c_3}) \in M$. In similar fashion we may confirm that each $r^a_{s_3}$ $(1 \leq a < n_3)$ is assigned to the hospital $h^{a+1}_{c_3}$ in first place on its preference list.

Now consider $r^{n_3}_{s_3}$. If the instance contains exactly three couples then the hospital in first place in the preference list of $r^{n_3}_{s_3}$ must be $h^0_{c_1}$. However, by construction, $(r^2_{c_1}, h^0_{c_1}) \in M$, a contradiction. Hence, the instance must have more than three couples and the hospital in first place in the preference list of $r^{n_3}_{s_3}$ must be a new hospital $h^{n_3+1}_{c_3}$. Now let the next couple be $(r^1_{c_4}, r^2_{c_4})$. Since $(r^{n_3}_{s_3}, h^{n_3+1}_{c_3}) \in M$, $(r^1_{c_4}, r^2_{c_4})$ cannot be assigned in M. Thus, whether $n_3 = 0$ or $n_3 > 0$, if $(r^1_{c_3}, r^2_{c_3})$ is assigned in M then $(r^1_{c_4}, r^2_{c_4})$ is not assigned in M.

Finally we consider whether $n_4 = 0$ or $n_4 > 0$. If $n_4 = 0$ and the instance contains exactly four couples then $(r^1_{c_4}, r^2_{c_4})$ expresses a preference for the hospital pair $(h^1_{c_4}, h^0_{c_1})$ and $h^0_{c_1}$ has $r^2_{c_4}$ in second place on its preference list and the result follows. Otherwise the instance contains more than four couples and $(r^1_{c_5}, r^2_{c_5})$ expresses a preference for $(h^1_{c_4}, h^1_{c_5})$. Now assume, $h^1_{c_4}$ is unassigned in M. Then $(r^1_{c_4}, r^2_{c_4})$ blocks M with $(h^{n_4+1}_{c_4}, h^1_{c_4})$, a contradiction. Thus $(r^1_{c_5}, r^2_{c_5})$ must be assigned to $(h^1_{c_4}, h^1_{c_5})$ in M.

If $n_4 > 0$ then $h^1_{c_4}$ has $r^1_{s_4}$ in first place on its preference list. If $r^1_{s_4}$ is not assigned in M then $(r^1_{s_4}, h^1_{c_4})$ blocks M, a contradiction. Hence $r^1_{s_4}$ must be assigned in M and moreover $(r^1_{s_4}, h^2_{c_4}) \in M$. In similar fashion we may confirm that each $r^a_{s_4}$ $(1 \leq a \leq n_4)$ must be assigned in M to the hospital $h^{a+1}_{s_4}$ in first place in its preference list. Now consider, $r^{n_4}_{s_4}$. If the instance contains exactly four couples then the hospital in first place in the preference list of $r^{n_4}_{s_4}$ must be $h^0_{c_1}$ and the result follows.

At this point we observe that argument is similar for the case that the number of couples is larger than four. As the preceding argument shows, if the number of couples is odd, then no stable matching exists, a contradiction.

Case (ii): Now suppose that $(r^1_{c_1}, r^2_{c_1})$ is unassigned in M. Then essentially $(r^1_{c_1}, r^2_{c_1})$ plays the role of $(r^1_{c_2}, r^2_{c_2})$ in the proof above and we may continue to generate a sequence of couples, every second of which is unassigned in M. Again, the same proof above can be used to infer that if the number of couples is odd, then no stable matching can exist.

Conversely, we show that if the number of couples in I is even then I admits a stable matching. For ease of exposition we use the description of the instance I shown in Figure 10 for this part of the proof. For clarity, this instance is exactly equivalent to the instance shown in Figure 9. Let M be the following matching in I where $h_{c_N}^{n_N+1} = h_{c_1}^0$ if $n_N > 0$ and $h_{c_N}^1 = h_{c_1}^0$ if $n_N = 0$:

$$M = \{(r_{c_1}^1, h_{c_1}^0), (r_{c_1}^2, h_{c_1}^1)\}$$
$$\bigcup \{(r_{c_k}^1, h_{c_{k-1}}^{n_{k-1}+1}), (r_{c_k}^2, h_{c_k}^1) : 2 \leq k \leq N,\ n_{k-1} > 0, k \bmod 2 \neq 0\}$$
$$\bigcup \{(r_{c_k}^1, h_{c_{k-1}}^2), (r_{c_k}^2, h_{c_k}^1) : 2 \leq k \leq N,\ n_{k-1} = 0, k \bmod 2 \neq 0\}$$
$$\bigcup \{(r_{s_k}^a, h_{c_k}^{a+1}) : 1 \leq k \leq N, 1 \leq a \leq n_k, n_k > 0\}$$

Assume M is unstable. Then there must exist a blocking pair of M in I.

Clearly no single resident $r_{s_k}^a$ $(1 \leq k \leq N, 1 \leq a \leq n_k, n_k > 0)$ can form part of a blocking pair for M in I as he is assigned in M to his first preference. Further, no couple $(r_{c_k}^1, r_{c_k}^2)$ $(2 \leq k \leq N, k \bmod 2 \neq 0\}$ can form part of a blocking pair for M in I as they are assigned to the hospital pair in first place on their joint preference list, $(h_{c_{k-1}}^{n_{k-1}+1}, h_{c_k}^1)$ if $n_k > 0$ or $(h_{c_{k-1}}^2, h_{c_k}^1)$ if $n_k = 0$.

Now, assume that $(r_{c_k}^1, r_{c_k}^2)$ $(2 \leq k \leq N, k \bmod 2 = 1)$ blocks M. If $n_{k-1} > 0$ then $(r_{c_k}^1, r_{c_k}^2)$ blocks with $(h_{c_{k-1}}^{n_{k-1}+1}, h_{c_k}^1)$. However, $h_{c_k}^1$ is assigned in M to its first preference $r_{s_k}^1$ and so cannot form part of a blocking pair, a contradiction. If $n_k = 0$ then $(r_{c_k}^1, r_{c_k}^2)$ blocks M with $(h_{c_{k-1}}^2, h_{c_k}^1)$. However, $h_{c_k}^1$ is assigned in M to its first preference (either $r_{s_k}^1$ if $n_k > 0$, or $r_{c_{k+1}}^1$ if $n_k = 0$) and so cannot form part of a blocking pair, a contradiction. Since no other possible blocking pairs exist for M in I it must be the case that M is a stable matching in I and the result is proven. □

Lemmas 12 and 13 lead to the following conclusion.

Theorem 14 $(2,1,2)$-MIN BP HRC *is solvable in polynomial time.*

Proof. Let I be an instance of $(2,1,2)$-HRC, and assume that M_0 is a matching in I in which all fixed assignments have been iteratively satisfied, and assume that the corresponding deletions have been made from the preference lists in I, yielding instance I'. Lemma 12 shows that I' is a union of sub-instances $I_1, I_2, \ldots, I_t$, where each I_j is of the form shown in Figure 9 $(1 \leq j \leq t)$.

For each j $(1 \leq j \leq t)$, we show how to construct a matching M_j in sub-instance I_j such that $|bp(M_j)| \leq 1$. Let N be the number of couples in I_j. Suppose firstly that N is even. The proof of Lemma 13 shows how to construct a matching M_j that is stable in I_j.

Now suppose that N is odd. Then $N \geq 3$, since all fixed assignments in I have been iteratively satisfied. By Lemma 13, I_j does not admit a stable matching; we will construct a matching M_j in I such that $|bp(M_j)| = 1$. Let k $(1 \leq k \leq N)$ be given.

Firstly assume that k is odd and $k \neq N$. Match $(r_{c_k}^1, r_{c_k}^2)$ to the hospital pair on their list. If $n_k > 0$, match $r_{s_k}^i$ to his first-choice hospital $h_{c_k}^{i+1}$ $(1 \leq i \leq n_k)$. Now assume that k is even. Leave $(r_{c_k}^1, r_{c_k}^2)$ unassigned. If $n_k > 0$, match $r_{s_k}^i$ to his second-choice hospital $h_{c_k}^i$ $(1 \leq i \leq n_k)$. Finally assume that $k = N$. If $n_N = 0$, leave couple $(r_{c_N}^1, r_{c_N}^2)$ unassigned. Otherwise match $(r_{c_N}^1, r_{c_N}^2)$ to the

hospital pair on their list. Also for each i $(1 \leq i \leq n_N - 1)$, match $r^i_{s_N}$ to his first-choice hospital $h^{i+1}_{c_N}$, and leave $r^{n_N}_{s_N}$ unassigned.

It is straightforward to verify that if $n_N > 0$ then $bp(M_j) = \{(r^{n_N}_{s_N}, h^{n_N}_{c_N})\}$ in I_j. Otherwise if $n_N = 0$ and $n_{N-1} = 0$, the only blocking pair of M_j in I_j involves the couple $(r^1_{c_{N-1}}, r^2_{c_{N-1}})$ and the hospital pair $(h^1_{c_{N-2}}, h^1_{c_{N-1}})$. Finally if $n_N = 0$ and $n_{N-1} > 0$, $bp(M_j) = \{(r^{n_{N-1}}_{s_{N-1}}, h^{n_{N-1}+1}_{c_{N-1}})\}$ in I_j.

Clearly $M = \cup^t_{j=0} M_j$ is then a most-stable matching in I. □

D An Integer Programming formulation for MIN BP HRC

D.1 Introduction

In this section we describe in detail our IP model for MIN BP HRC. Let I be an instance of HRC; we will denote by J the IP model corresponding to I. We describe the variables, constraints and objective function for J in Sections D.2, D.3 and D.4 respectively. In Section D.3, the text in bold before the definition of a constraint shows the blocking pair type from Definition 1 to which the constraint corresponds. Finally in Section D.5 we present a proof of correctness for the IP model for MIN BP HRC. In the remainder of this section we assume the notation defined at the beginning of Section 3.

D.2 Variables in the IP model

In J, for each i $(1 \leq i \leq n_1)$ and p $(1 \leq p \leq l(r_i))$, define a variable $x_{i,p}$ such that

$$x_{i,p} = \begin{cases} 1 \text{ if } r_i \text{ is assigned to his } p^{th} \text{ choice hospital} \\ 0 \text{ otherwise.} \end{cases}$$

For $p = l(r_i)+1$ define a variable $x_{i,p}$ whose intuitive meaning is that resident r_i is unassigned. Thus we also have that

$$x_{i,l(r_i)+1} = \begin{cases} 1 \text{ if } r_i \text{ is unassigned} \\ 0 \text{ otherwise.} \end{cases}$$

Let $X = \{x_{i,p} : 1 \leq i \leq n_1, 1 \leq p \leq l(r_i)+1\}$. For ease of exposition we define some additional notation. For each i $(1 \leq i \leq c)$ and p $(1 \leq p \leq l(r_{2i-1}))$, let $pref((r_{2i-1}, r_{2i}), p)$ denote the hospital pair at position p on the joint preference list of (r_{2i-1}, r_{2i}).

Now, for all j $(1 \leq j \leq n_2)$ and q $(1 \leq q \leq l(h_j))$, define a new variable $\alpha_{j,q} \in \{0, 1\}$. The intuitive meaning of a variable $\alpha_{j,q}$ is that if h_j is fully subscribed with assignees better than rank q then $\alpha_{j,q}$ may take the value 0 or 1. However, if h_j is not full with assignees better than rank q then $\alpha_{j,q} = 1$. Constraints (2) and 13 described in Section D.3 are applied to enforce this property.

Now, for all j $(1 \leq j \leq n_2)$ and q $(1 \leq q \leq l(h_j))$, define a new variable $\beta_{j,q} \in \{0, 1\}$. The intuitive meaning of a variable $\beta_{j,q}$ is that if h_j has $c_j - 1$ or

more assignees better than rank q then $\beta_{j,q}$ may take a value of 0 or 1. However, if h_j has fewer than $c_j - 1$ assignees better than rank q then $\beta_{j,q} = 1$. Constraints (3) and 14 described in Section D.3 are applied to enforce this property.

Finally, for all i $(1 \leq i \leq n_1)$ and p $(1 \leq p \leq l(r_i))$, define a new variable $\theta_{i,p} \in \{0, 1\}$. The intuitive meaning of a variable $\theta_{i,p}$ is that $\theta_{i,p} = 1$ if resident r_i is involved in a blocking pair with the hospital at position p on his preference list, either as a single resident or as part of a couple, and $\theta_{i,p} = 0$ otherwise.

D.3 Constraints in the IP model

The following constraint simply ensures that each variable $x_{i,p}$ must be binary valued for all i $(1 \leq i \leq n_1)$ and p $(1 \leq p \leq l(r_i) + 1)$:

$$x_{i,p} \in \{0, 1\} \tag{1}$$

Similarly, the following constraint ensures that each variable $\alpha_{j,q}$ must be binary valued for all j $(1 \leq j \leq n_2)$ and q $(1 \leq q \leq l(h_j))$:

$$\alpha_{j,q} \in \{0, 1\} \tag{2}$$

Also, the following constraint ensures that each variable $\beta_{j,q}$ must be binary valued for all j $(1 \leq j \leq n_2)$ and q $(1 \leq q \leq l(h_j))$:

$$\beta_{j,q} \in \{0, 1\} \tag{3}$$

Similarly the following constraint ensures that each variable $\theta_{i,p}$ must be binary valued for all i $(1 \leq i \leq n_1)$ and p $(1 \leq p \leq l(r_i) + 1)$:

$$\theta_{i,p} \in \{0, 1\} \tag{4}$$

As each resident $r_i \in R$ is assigned to exactly one hospital or is unassigned (but not both), we introduce the following constraint for all i $(1 \leq i \leq n_1)$:

$$\sum_{p=1}^{l(r_i)+1} x_{i,p} = 1 \tag{5}$$

Since a hospital h_j may be assigned at most c_j residents, $x_{i,p} = 1$ where $pref(r_i, p) = h_j$ for at most c_j residents. We thus obtain the following constraint for all j $(1 \leq j \leq n_2)$:

$$\sum_{i=1}^{n_1} \sum_{p=1}^{l(r_i)} \{x_{i,p} \in X : pref(r_i, p) = h_j\} \leq c_j \tag{6}$$

For each couple (r_{2i-1}, r_{2i}), r_{2i-1} is unassigned if and only if r_{2i} is unassigned, and r_{2i-1} is assigned to the hospital in position p in their individual list if and

only if r_{2i} is assigned to the hospital in position p in their individual list. We thus obtain the following constraint for all i $(1 \le i \le c)$ and p $(1 \le p \le l(r_{2i-1})+1)$:

$$x_{2i-1,p} = x_{2i,p} \tag{7}$$

Type 1 blocking pairs. In matching M in I, if a single resident $r_i \in R$ is unassigned or has a worse partner than some hospital $h_j \in H$ where $\mathit{pref}(r_i, p) = h_j$ and $\mathit{rank}(h_j, r_i) = q$ then h_j must be fully subscribed with better partners than r_i, for otherwise (r_i, h_j) blocks M. Hence if r_i is unassigned or has worse partner than h_j, i.e., $\sum_{p'=p+1}^{l(r_i)+1} x_{i,p'} = 1$, and h_j is not fully subscribed with better partners than r_i, i.e., $\sum_{q'=1}^{q-1} \{x_{i',p''} \in X : (r_{i'}, p'') \in R(h_j, q')\} < c_j$, then we require $\theta_{i,p} = 1$ to count this blocking pair. Thus, for each i $(2c+1 \le i \le n_1)$ and p $(1 \le p \le l(r_i))$ we obtain the following constraint where $\mathit{pref}(r_i, p) = h_j$ and $\mathit{rank}(h_j, r_i) = q$:

$$c_j \left(\left(\sum_{p'=p+1}^{l(r_i)+1} x_{i,p'} \right) - \theta_{i,p} \right) \le \sum_{q'=1}^{q-1} \{x_{i',p''} \in X : (r_{i'}, p'') \in R(h_j, q')\} \tag{8}$$

In this way, we can count the number of blocking pairs using the $\theta_{i,p}$ values. A similar methodology is used in all replacement constraints for the remaining stability criteria that follow. Ultimately, the number of blocking pairs is the sum of the $\theta_{i,p}$ values, except that to avoid counting a blocking pair twice in the case of a couple, the model will assume that $\theta_{2i,p} = 0$ for all i $(1 \le i \le c)$ and for all p $(1 \le p \le l(r_{2i}))$.

Type 2a blocking pairs. In a matching M in I, if a couple $\mathcal{C}_i = (r_{2i-1}, r_{2i})$ jointly prefer hospital pair (h_{j_1}, h_{j_2}), at position p_1 in $\mathcal{C}_i$'s joint preference list, to $(M(r_{2i-1}), M(r_{2i}))$, at position p_2, and h_{j_1} is undersubscribed or prefers r_{2i-1} to one of its assignees in M, and $h_{j_2} = M(r_{2i})$, then (r_{2i-1}, r_{2i}) blocks M with (h_{j_1}, h_{j_2}). In the special case where $\mathit{pref}(r_{2i-1}, p_1) = \mathit{pref}(r_{2i}, p_1) = h_{j_1}$, if $h_{j_1} = h_{j_2} = M(r_{2i})$, h_{j_1} is undersubscribed or prefers r_{2i-1} to one of its assignees in M other than r_{2i}, then again (r_{2i-1}, r_{2i}) blocks M with (h_{j_1}, h_{j_2}).

Thus, for the general case, we obtain the following constraint for all i $(1 \le i \le c)$ and p_1, p_2 $(1 \le p_1 < p_2 \le l(r_{2i-1}))$ such that $\mathit{pref}(r_{2i}, p_1) = \mathit{pref}(r_{2i}, p_2)$ and $\mathit{rank}(h_{j_1}, r_{2i-1}) = q$:

$$c_{j_1}(x_{2i-1,p_2} - \theta_{2i-1,p_1}) \le \sum_{q'=1}^{q-1} \{x_{i',p''} \in X : (r_{i'}, p'') \in R(h_{j_1}, q')\} \tag{9}$$

For the special case in which $\mathit{pref}(r_{2i-1}, p_1) = \mathit{pref}(r_{2i}, p_1) = h_{j_1}$ we obtain the following constraint for all i $(1 \le i \le c)$ and p_1, p_2 where $(1 \le p_1 < p_2 \le l(r_{2i-1}))$

such that $pref(r_{2i}, p_1) = pref(r_{2i}, p_2)$ and $rank(h_{j_1}, r_{2i-1}) = q$:

$$(c_{j_1} - 1)(x_{2i-1,p_2} - \theta_{2i-1,p_1}) \leq \sum_{q'=1}^{q-1} \{x_{i',p''} \in X : q' \neq rank(h_{j_1}, r_{2i}) \wedge (r_{i'}, p'') \in R(h_{j_1}, q')\} \quad (10)$$

Type 2b blocking pairs. A similar constraint is required for the case that the odd-subscript member of a given couple stays assigned to the same hospital. Thus, for the general case, we obtain the following constraint for all i ($1 \leq i \leq c$) and p_1, p_2 where ($1 \leq p_1 < p_2 \leq l(r_{2i})$) such that $pref(r_{2i-1}, p_1) = pref(r_{2i-1}, p_2)$ and $rank(h_{j_2}, r_{2i}) = q$:

$$c_{j_2}(x_{2i-1,p_2} - \theta_{2i-1,p_1}) \leq \sum_{q'=1}^{q-1} \{x_{i',p''} \in X : (r_{i'}, p'') \in R(h_{j_2}, q')\} \quad (11)$$

Again, for the special case in which $pref(r_{2i-1}, p_1) = pref(r_{2i}, p_1) = h_{j_2}$ we obtain the following constraint for all i ($1 \leq i \leq c$) and p_1, p_2 where ($1 \leq p_1 < p_2 \leq l(r_{2i})$) such that $pref(r_{2i-1}, p_1) = pref(r_{2i-1}, p_2)$ and $rank(h_{j_2}, r_{2i}) = q$:

$$(c_{j_1} - 1)(x_{2i-1,p_2} - \theta_{2i-1,p_1}) \leq \sum_{q'=1}^{q-1} \{x_{i',p''} \in X : q' \neq rank(h_{j_2}, r_{2i-1}) \wedge (r_{i'}, p'') \in R(h_{j_2}, q')\} \quad (12)$$

Now, we define a variable $\alpha_{j,q}$ such that if h_j is full with assignees better than rank q then $\alpha_{j,q}$ may take the value of zero or one. Otherwise, h_j is not full with assignees better than rank q and $\alpha_{j,q} = 1$. Hence, we obtain the following constraint for all j ($1 \leq j \leq n_2$) and q ($1 \leq q \leq l(h_j)$):

$$\alpha_{j,q} \geq 1 - \frac{\sum_{q'=1}^{q-1} \{x_{i,p} \in X : (r_i, p) \in R(h_j, q')\}}{c_j} \quad (13)$$

Next we define a variable $\beta_{j,q}$ such that if h_j has $c_j - 1$ or more assignees better than rank q then $\beta_{j,q}$ may take a value of zero or one. Otherwise, h_j has fewer than $c_j - 1$ assignees better than rank q and $\beta_{j,q} = 1$. Hence, we obtain the following constraint all j ($1 \leq j \leq n_2$) and q ($1 \leq q \leq l(h_j)$):

$$\beta_{j,q} \geq 1 - \frac{\sum_{q'=1}^{q-1} \{x_{i,p} \in X : (r_i, p) \in R(h_j, q')\}}{(c_j - 1)} \quad (14)$$

Type 3a blocking pairs. In a matching M in I, if a couple $\mathcal{C}_i = (r_{2i-1}, r_{2i})$ is unassigned or assigned to a worse hospital pair than (h_{j_1}, h_{j_2}) (where $h_{j_1} \neq$

h_{j_2}), and for each $t \in \{1, 2\}$, h_{j_t} is undersubscribed and finds r_{2i-2+t} acceptable, or prefers r_{2i-2+t} to its worst assignee, then (r_{2i-1}, r_{2i}) blocks M with (h_{j_1}, h_{j_2}). Thus we obtain the following constraint for all i $(1 \leq i \leq c)$ and p $(1 \leq p \leq l(r_{2i-1}))$ where $h_{j_1} = \mathit{pref}(r_{2i-1}, p)$, $h_{j_2} = \mathit{pref}(r_{2i}, p)$, $h_{j_1} \neq h_{j_2}$, $\mathit{rank}(h_{j_1}, r_{2i-1}) = q_1$ and $\mathit{rank}(h_{j_2}, r_{2i}) = q_2$:

$$\sum_{p'=p+1}^{l(r_{2i-1})+1} x_{2i-1,p'} + \alpha_{j_1,q_1} + \alpha_{j_2,q_2} - \theta_{2i-1,p} \leq 2 \tag{15}$$

Type 3b/c blocking pairs. In a matching M in I, if a couple $\mathcal{C}_i = (r_{2i-1}, r_{2i})$ is unassigned or assigned to a worse pair than (h_j, h_j) where $M(r_{2i-1}) \neq h_j$ and $M(r_{2i}) \neq h_j$, (r_{2i-1}, r_{2i}) finds (h_j, h_j) acceptable, and h_j has two or more free posts available, then (r_{2i-1}, r_{2i}) blocks M with (h_j, h_j) – this is a Type 3b blocking pair. In a matching M in I, if a couple $\mathcal{C}_i = (r_{2i-1}, r_{2i})$ is unassigned or assigned to a worse pair than (h_j, h_j) where $M(r_{2i-1}) \neq h_j$ and $M(r_{2i}) \neq h_j$, (r_{2i-1}, r_{2i}) finds (h_j, h_j) acceptable, and h_j prefers at least one of r_{2i-1} or r_{2i} to some assignee of h_j in M while simultaneously having a single free post, then (r_{2i-1}, r_{2i}) blocks M with (h_j, h_j) – this is a Type 3c blocking pair.

These two blocking pair types may be modelled by a single constraint. For each i $(1 \leq i \leq c)$ and p $(1 \leq p \leq l(r_{2i-1}))$ such that $\mathit{pref}(r_{2i-1}, p) = \mathit{pref}(r_{2i}, p)$ and $h_j = \mathit{pref}(r_{2i-1}, p)$, where $q = \min\{\mathit{rank}(h_j, r_{2i}),\ \mathit{rank}(h_j, r_{2i-1})\}$, we enforce the following:

$$c_j \left(\left(\sum_{p'=p+1}^{l(r_{2i-1})+1} x_{2i-1,p'} \right) - \theta_{2i-1,p} \right) - \frac{\sum_{q'=1}^{q-1} \{x_{i',p''} \in X : (r_{i'}, p'') \in R(h_j, q')\}}{(c_j - 1)}$$

$$\leq \sum_{q'=1}^{l(h_j)} \{x_{i',p''} \in X : (r_{i'}, p'') \in R(h_j, q')\} \tag{16}$$

Type 3d blocking pairs. In a matching M in I, if a couple $\mathcal{C}_i = (r_{2i-1}, r_{2i})$ is unassigned or jointly assigned to a worse pair than (h_j, h_j) where $M(r_{2i-1}) \neq h_j$ and $M(r_{2i}) \neq h_j$, and h_j is full and also has two assignees r_s and r_t (where $s \neq t$) such that h_j prefers r_{2i-1} to r_s and h_j prefers r_{2i} to r_t, then (r_{2i-1}, r_{2i}) blocks M with (h_j, h_j).

For each (h_j, h_j) acceptable to (r_{2i-1}, r_{2i}), let r_{min} be the better of r_{2i-1} and r_{2i} according to hospital h_j with $\mathit{rank}(h_j, r_{min}) = q_{min}$. Analogously, let r_{max} be the worse of r_{2i} and r_{2i-1} according to hospital h_j with $\mathit{rank}(h_j, r_{max}) = q_{max}$. Then we obtain the following constraint for i $(1 \leq i \leq c)$ and p $(1 \leq p \leq l(r_{2i-1}))$ such that $\mathit{pref}(r_{2i-1}, p) = \mathit{pref}(r_{2i}, p) = h_j$:

$$\sum_{p'=p+1}^{l(r_{2i-1})+1} x_{2i-1,p'} + \alpha_{j,q_{max}} + \beta_{j,q_{min}} - \theta_{2i-1,p} \leq 2 \tag{17}$$

D.4 Objective function in the IP model

A maximum cardinality most-stable matching M is a matching of maximum cardinality, taken over all most-stable matchings in I. To compute such a matching in J, we apply two objective functions in sequence.

First we find an optimal solution in J that minimises the number of blocking pairs. To this end, we apply the following objective function:

$$\min \sum_{i=1}^{n_1} \sum_{p=1}^{l(r_i)} \theta_{i,p} \tag{18}$$

The matching M corresponding to an optimal solution in J will be a most-stable matching in I. Let $k = |bp(M)|$. Now we week a maximum cardinality matching in I with at most k blocking pairs. Thus we add the following constraint to J, which ensures that, when maximising on cardinality, any solution also has at most k blocking pairs:

$$\sum_{i=1}^{n_1} \sum_{p=1}^{l(r_i)} \theta_{i,p} \leq k \tag{19}$$

The final step is to maximise the size of the matching, subject to the matching being most-stable. This involves optimising for a second time, this time using the following objective function:

$$\max \sum_{i=1}^{n_1} \sum_{p=1}^{l(r_i)} x_{i,p}. \tag{20}$$

D.5 Proof of correctness for the IP model

We now establish the correctness of the IP model for MIN BP HRC presented in Sections D.2, D.3 and D.4.

Theorem 15 *Given an instance I of* MIN BP HRC*, let J be the corresponding IP model as defined in Sections D.2, D.3 and D.4 (omitting Constraint* (19) *and objective function* (20)*). A most-stable matching in I is exactly equivalent to an optimal solution to J with respect to objective function* (18).

Proof. Let M be a most-stable matching in I. Let $\langle \mathbf{x}, \boldsymbol{\alpha}, \boldsymbol{\beta} \rangle$ be the corresponding assignment of boolean values to the variables in J as constructed in the proof of Theorem 12 in [46]. Initially let $\theta_{i,p} = 0$ for all i $(1 \leq i \leq n_1)$ and p $(1 \leq p \leq l(r_i))$.

Assume that (r_i, h_j) blocks M where r_i is a single resident and $pref(r_i, p) = h_j$. Then Constraint (8) will be violated if $\theta_{i,p} = 0$. Set $\theta_{i,p} = 1$. Then the LHS of Constraint (8) becomes 0 and the constraint is satisfied.

Now, assume that (r_i, r_j) blocks M with (h_k, h_l) for some couple (r_i, r_j), where $pref((r_i, r_j), p) = (h_k, h_l)$. Then depending on which part of Definition

1 is violated, one of the constraints in the range (9)-(12) and (15)-(17) will be violated if $\theta_{i,p} = 0$. By setting $\theta_{i,p} = 1$, the constraint concerned will be satisfied.

It follows that $\langle \mathbf{x}, \boldsymbol{\alpha}, \boldsymbol{\beta}, \boldsymbol{\theta} \rangle$ is a feasible solution to J. Moreover the objective value of this solution is equal to $|bp(M)|$.

Conversely, let $\langle \mathbf{x}, \boldsymbol{\alpha}, \boldsymbol{\beta}, \boldsymbol{\theta} \rangle$ be an optimal solution to J and let M be the corresponding matching in I as constructed in the proof of Theorem 12 in [46].

Now assume that $\theta_{i,p} = 1$ for some i $(1 \leq i \leq n_1)$ and p $(1 \leq p \leq l(r_i))$. If r_i is not involved in a blocking pair with h_j where $pref(r_i, p) = h_j$ (either as a single resident or part of a couple), then by Theorem 12 in [46], Constraints (8) to (12) and (15)-(17) are satisfied with $\theta_{i,p} = 0$, in contradiction to the fact that $\langle \mathbf{x}, \boldsymbol{\alpha}, \boldsymbol{\beta}, \boldsymbol{\theta} \rangle$ is optimal according to objective function (18). Thus if $\theta_{i,p} = 1$ for some i $(1 \leq i \leq n_1)$ and p $(1 \leq p \leq l(r_{n_1}))$ then r_i must be involved in a blocking pair with the hospital in position p on his preference list.

On the other hand, by the first direction, if there is a blocking pair of M, there must be a unique corresponding $\theta_{i,p}$ that has value 1. It follows that $|bp(M)|$ is equal to the objective value of $\langle \mathbf{x}, \boldsymbol{\alpha}, \boldsymbol{\beta}, \boldsymbol{\theta} \rangle$ in J. □

By enforcing Constraint (19) and imposing objective function (20), we obtain the following corollary.

Corollary 16 *Given an instance I of* MIN BP HRC *let J be the corresponding IP model as defined in Sections D.2, D.3 and D.4 (omitting objective function* (18)*). A maximum cardinality most-stable matching in I is exactly equivalent to an optimal solution to J with respect to objective function* (20).

References

43. J. Drummond, A. Perrault, and F. Bacchus. SAT is an effective and complete method for solving stable matching problems with couples. In *Proceedings of IJCAI '15: the Twenty-Fourth International Joint Conference on Artificial Intelligence*, pages 518–525. AAAI Press, 2015.
44. M.R. Garey, D.S. Johnson, and L. Stockmeyer. Some simplified NP-complete graph problems. *Theoretical Computer Science*, 1:237–267, 1976.
45. D. Maier and J.A. Storer. A note on the complexity of the superstring problem. Technical Report 233, Princeton University, Department of Electrical Engineering and Computer Science, Princeton, NJ, October 1977.
46. P. Biró, D.F. Manlove, and I. McBride. The Hospitals / Residents problem with Couples: Complexity and integer programming models. Technical Report arXiv:1308.4534, Computing Research Repository, Cornell University Library, 2013. Available from `http://arxiv.org/abs/1308.4534`.
